# Targeting nucleotide metabolism enhances the efficacy of anthracyclines and anti-metabolites in triple-negative breast cancer

**DOI:** 10.1038/s41523-021-00245-5

**Published:** 2021-04-06

**Authors:** Craig Davison, Roisin Morelli, Catherine Knowlson, Melanie McKechnie, Robbie Carson, Xanthi Stachtea, Kylie A. McLaughlin, Vivien E. Prise, Kienan Savage, Richard H. Wilson, Karl A. Mulligan, Peter M. Wilson, Robert D. Ladner, Melissa J. LaBonte

**Affiliations:** 1 Medicine, Dentistry and Biomedical Sciences: Patrick G Johnston Centre for Cancer Research, Queen’s University Belfast, Belfast, UK; 2 CV6 Therapeutics (NI) Ltd, Belfast, UK; 3 Translational Research Centre, University of Glasgow, Glasgow, UK; 4 Present Address: Medicine, Dentistry and Biomedical Sciences: Patrick G Johnston Centre for Cancer Research, Queen’s University Belfast, Belfast, UK

**Keywords:** Breast cancer, Base excision repair

## Abstract

Triple-negative breast cancer (TNBC) remains the most lethal breast cancer subtype with poor response rates to the current chemotherapies and a lack of additional effective treatment options. We have identified deoxyuridine 5′-triphosphate nucleotidohydrolase (dUTPase) as a critical gatekeeper that protects tumour DNA from the genotoxic misincorporation of uracil during treatment with standard chemotherapeutic agents commonly used in the FEC regimen. dUTPase catalyses the hydrolytic dephosphorylation of deoxyuridine triphosphate (dUTP) to deoxyuridine monophosphate (dUMP), providing dUMP for thymidylate synthase as part of the thymidylate biosynthesis pathway and maintaining low intracellular dUTP concentrations. This is crucial as DNA polymerase cannot distinguish between dUTP and deoxythymidylate triphosphate (dTTP), leading to dUTP misincorporation into DNA. Targeting dUTPase and inducing uracil misincorporation during the repair of DNA damage induced by fluoropyrimidines or anthracyclines represents an effective strategy to induce cell lethality. dUTPase inhibition significantly sensitised TNBC cell lines to fluoropyrimidines and anthracyclines through imbalanced nucleotide pools and increased DNA damage leading to decreased proliferation and increased cell death. These results suggest that repair of treatment-mediated DNA damage requires dUTPase to prevent uracil misincorporation and that inhibition of dUTPase is a promising strategy to enhance the efficacy of TNBC chemotherapy.

## Introduction

Triple-negative breast cancer (TNBC) is an aggressive breast cancer subtype that lacks oestrogen receptor, progesterone receptor, and human epidermal receptor 2^1^ and is associated with an overall poor prognosis^2,3^. At present, the treatment of TNBC lacks any effective targeted therapies, leaving chemotherapy regimens that incorporate anthracycline and taxane-based therapeutics as the standard of care (SoC). While 30% of patients respond well to chemotherapy, the remaining patients have limited improvements in clinical outcomes, highlighting the critical need for effective therapeutic strategies to treat TNBC^4^.

While clinical trials for immunotherapy, checkpoint inhibition, antibody-drug conjugates, and other promising agents are under investigation in the evolving treatment landscape, this study focuses on the identification of metabolic vulnerabilities in TNBC that represent potential new therapeutic strategies to improve SoC anthracycline/taxane-based chemotherapy for early TNBC and anthracycline or anti-metabolite-based treatments for metastatic TNBC.

The metabolic landscape of TNBC is typified by significant intrinsic heterogeneity in cellular metabolism that drives proliferation, survival, and metastasis^1,5,6^. The targeting of aberrant metabolic pathways may identify vulnerabilities that can be therapeutically targeted to disrupt the biosynthesis and processing of key macromolecules including proteins, lipids, or nucleotides^7,8^. Beyond intrinsic metabolic alterations, adaptive metabolic reprogramming has been characterized in TNBC following genotoxic chemotherapy with doxorubicin and identified de novo pyrimidine synthesis as a promoter of therapy resistance^6^.

In this study, we sought to identify and characterize new therapeutic opportunities to enhance current SoC chemotherapies that incorporate anthracyclines and the anti-metabolite 5-Fluorouracil (5-FU) in TNBC through further modulation of pyrimidine and uracil nucleotide metabolism pathways. We hypothesized that this could be achieved through inhibition of the gatekeeper enzyme, deoxyuridine 5′-triphosphate nucleotidohydrolase (dUTPase), as this enzyme functions to prevent uracil misincorporation into DNA^9^.

5-FU exerts its anticancer activity via the inhibition of the pyrimidine biosynthetic enzyme thymidylate synthase (TS), inducing a metabolic blockade that results in the acute depletion of thymidylate and subsequently dTTP which is required for DNA synthesis and repair. This results in rapid growth arrest, and in some instances of prolonged dTTP depletion, and tumour cell lethality. Inhibition of TS also results in the accumulation of the TS enzyme-substrate dUMP, which, upon further phosphorylation by ubiquitous pyrimidine monophosphate and diphosphate kinases, can lead to rapid and abnormal expansion of the deoxyuridine triphosphate (dUTP) pool. DNA polymerases cannot distinguish between dUTP and dTTP and will incorporate either substrate into DNA. Therefore, as TS inhibition has depleted cells of dTTP, the abnormal and unusually high availability of dUTP leads to its misincorporation into newly synthesized DNA as uracil^10^. As uracil is not a native component of DNA, the base excision repair (BER) pathway attempts to remove the misincorporated uracil. However, as dTTP levels remain low due to TS inhibition and dUTP pools remain high, futile cycles of uracil misincorporation, failed repair and further misincorporation ensue resulting in catastrophic DNA damage and cancer cell death. DNA damage due to misincorporation of dUTP is highly dependent on the levels of the enzyme dUTPase, which rapidly degrades dUTP to dUMP (and PPi) and, therefore prevents dUTP pool accumulation, protecting tumour DNA from the lethal effects of uracil misincorporation during TS inhibition. Our group and others have demonstrated conclusively that targeting dUTPase sensitises cancer cells to TS-targeting therapies^10–14^. In addition, recent research has also demonstrated that causing uracil misincorporation at sites of DNA damage repair can sensitise cancer cells to anthracyclines^15,16^. Based on these observations, this study sought to explore the potential application of promising combination strategies in TNBC.

## Results

### Inhibition of dUTPase sensitises TNBC cells to fluoropyrimidine chemotherapy

To examine the potential of inducing uracil DNA misincorporation as an effective therapeutic strategy to enhance TNBC response to TS inhibitors and anthracyclines, the catalytic activity of dUTPase was blocked, either by small interfering RNA (siRNA) or by small-molecule inhibition and the impact on cancer cell proliferation and survival in combination with anthracyclines or TS inhibitors was evaluated.

**Fig. 1:**
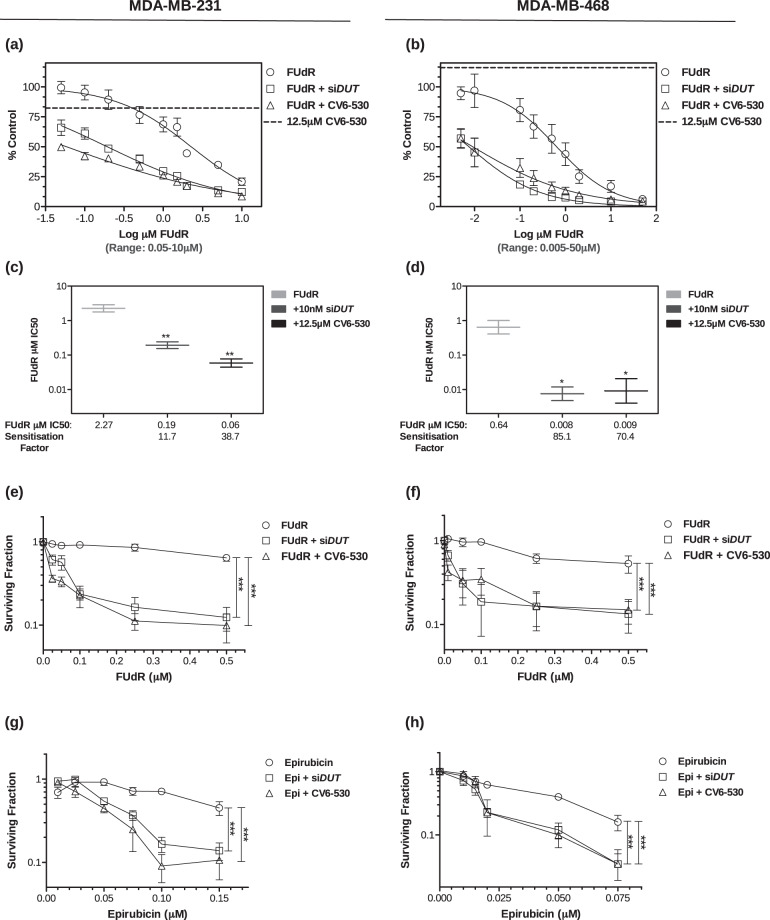
Inhibition of dUTPase sensitises TNBC cells to fluoropyrimidine and anthracycline chemotherapies. MDA-MB-231 and MDA-MB-468 cells were analysed for (**a**, **b**) cell viability following dUTPase inhibition (10 nM si*DUT* or 12.5 µM CV6-530) in combination with 0.05–10 µM or 0.005-50 µM FUdR for 96 h and quantified with CellTiter-Glo assay (Promega). The single-agent growth inhibitory effect of 12.5 µM CV6-530 is represented by a horizontal dotted line on both graphs to allow comparisons for each combination. Data points represent mean ± SEM percent growth inhibition (*N* = 3) compared with vehicle-treated controls at 100%. **c**, **d** IC_50_ values were calculated using Graphpad (Prism) and sensitisation factors were calculated by dividing the single-agent FUdR IC_50_ by the IC_50_ of the combination of FUdR and 12.5 µM CV6-530. **e**–**h** Cell survival was determined by colony-forming capacity, where MDA-MB-231 and MDA-MB-468 cells were treated with either (**e**, **f**) 0.025–0.5 µM FUdR for 24 h or (**g**) 0.01–0.1 µM epirubicin (Epi) in MDA-MB-231 and (**h**) 0-0.075 µM Epi in MDA-MB-468 for 4 h in combination with 12.5 µM CV6-530 for 24 h. The media was subsequently replaced with drug-free media for 12–15 days and cells were allowed to form colonies (>50 cells). Data are presented as the mean ± SEM percentage colony formation compared with vehicle-treated control (*N* = 3, independent experiments). Statistical analysis determined by an unpaired, two-tailed Student *t*-test. ns, not significant; *P < 0.05; **P < 0.01; ***P < 0.001.

Silencing of dUTPase was achieved by pre-treating cells with 10 nM si*DUT* which resulted in a reduction in dUTPase mRNA of 89% and 85% in MDA-MB-231 and MDA-MB-468 cells, respectively, and a decrease in dUTPase protein of >80% in both cell lines. Small molecule inhibition of dUTPase was achieved using CV6-530, which inhibits dUTPase enzymatic activity with an IC_50_ of 359 nMol/L (Supplementary Fig. 1A–C). The inhibition of dUTPase alone had no significant impact on cell viability or survival in MDA-MB-231, MDA-MB-468, or CAL51 cells treated with 12.5μM CV6-530 (Supplementary Fig. 1D–F). However, when either si*DUT* or CV6-530 was combined with FUdR, highly significant decreases in cell proliferation and survival compared to single-agent FUdR were observed across all FUdR concentrations (Fig. 1A–F). The addition of exogenous thymidine (allowing dTTP production via the salvage pathway) completely rescued the treatment-related effects on cell survival, demonstrating the effects were dependent on dTTP pool depletion (Supplementary Fig. 2).

### Inhibition of dUTPase sensitises TNBC cells to anthracycline chemotherapy

Inhibition of dUTPase in combination with TS inhibitors has been well established to show anticancer activity across multiple cancer types^10–14^. However, recent research has demonstrated that anthracycline treatment induces increased de novo nucleotide synthesis required to repair DNA damage which is a vulnerability that can be exploited^6^. Inhibition of dUTPase by both si*DUT* and CV6-530 also sensitised the MDA-MB-231 and MDA-MB-468 cell lines to the anthracyclines, epirubicin (Fig. 1G, H) and doxorubicin (Supplementary Fig. 3), but not to the platinum agent’s cisplatin or carboplatin (Supplementary Fig. 3). In the MDA-MB-231, there was a significant reduction in survival following epirubicin or doxorubicin with dUTPase inhibition (DUTi) by 61.9% ± 7.3 (*P* < 0.0001) and 42.9% ± 5.4 (*P* < 0.0001), respectively, compared to 0.075 µM single agents. Interestingly, this sensitisation was limited to survival, with no significant sensitization observed in the short-term growth inhibition/viability assay suggesting that the combination does not increase the initial short-term growth inhibitory effects from the anthracycline-induced DNA damage, but perhaps impairs DNA repair and cell recovery (Supplementary Fig. 3).

### Inhibition of dUTPase enhances DNA damage and cell death induced by fluoropyrimidine and anthracycline chemotherapies

**Fig. 2:**
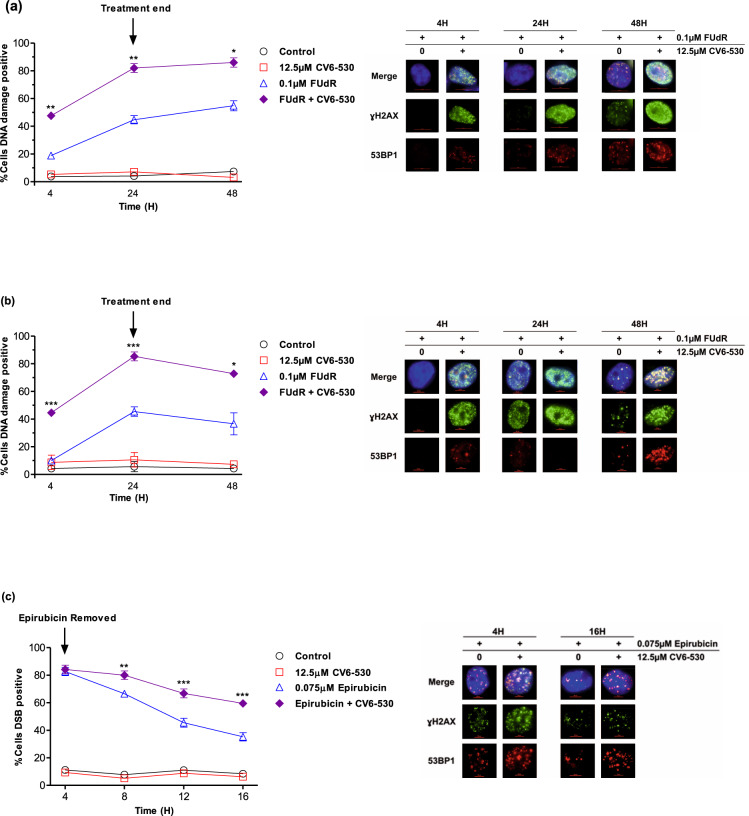
Inhibition of dUTPase enhances DNA damage induced by fluoropyrimidine and anthracycline chemotherapies. **a** MDA-MB-231 and **b** MDA-MB-468 cells were treated with 0.1 µM FUdR or 12.5 µM CV6-530 alone or in combination for 24 h when the media was replaced with drug-free media. At 4, 24, and 48 h, cells were fixed, stained, and imaged for ɣH2A.X (green) and 53BP1 (red). Line graphs represent the mean ± SEM percentage of cells positive for DNA damage (ɣH2A.X) or double-strand breaks (>5 co-localised ɣH2A.X/53BP1 foci) quantified from *N* = 3 independent experiments. >100 cells were scored per experiment. Representative immunofluorescent images of ɣH2A.X and 53BP1 marked DNA damage are shown on the right. **c** MDA-MB-231 cells were treated with 0.075 µM epirubicin for 4 h or 12.5 µM CV6-530 for 16 h alone or in combination. At 4, 8, 12 and 16 h, cells were fixed, stained and imaged for ɣH2A.X (green) and 53BP1 (red). Line graphs represent the mean ± SEM percentage cells positive for DNA damage (ɣH2A.X) or double-strand breaks (>5 co-localised ɣH2A.X/53BP1 foci) quantified from *N* = 3 independent experiments. >100 cells were scored per experiment Representative images of γ-H2AX and 53BP1 are shown on the right.

FUdR induced inhibition of de novo pyrimidine synthesis efficiently blocks DNA synthesis and repair which results in DNA damage^10,17^. Anthracyclines-induced DNA double-strand breaks (DSBs) also require increasing de novo nucleotide production for efficient repair via new DNA synthesis^6^. We, therefore, investigated the impact of DUTi in combination with FUdR or epirubicin on the induction of DNA damage and subsequent repair. For FUdR, a low-dose (0.1 µM) was selected that alone showed no significant alteration of proliferation or survival, but in combination with CV6-530 significantly reduced survival both in MDA-MB-231 and MDA-MB-468 cells (Fig. 1). The combination in both cell lines resulted in a significant increase in DNA damage compared with single agents (as determined by the γH2AX foci) after 4 h (*P* = 0.001; *P* < 0.0001, respectively) that further increased at 24 h and remained elevated to 24 h post-drug removal (Fig. 2A, B). Epirubicin at 0.075 µM increased the number of DSB positive cells to 82.7% ± 3.0 (*P* < 0.0001) after a 4 h treatment, which was subsequently repaired and reduced to 47.4% (±4.3%) at 16 h. DUTi or *siDUT* in combination with epirubicin did not significantly alter the total number of DSB positive cells following 4 h epirubicin treatment (82.7% vs. 84.2%; *P* = 0.74), however, the combination resulted in a decrease in the repair of the DSB breaks out to 16 h (Fig. 2C**;** Supplementary Fig. 4) suggesting that the sensitisation caused by targeting dUTPase with anthracyclines is not due to changes in the initial DNA damage, but rather interferes with the subsequent DSB repair leading to the persistence of DSBs.

**Fig. 3:**
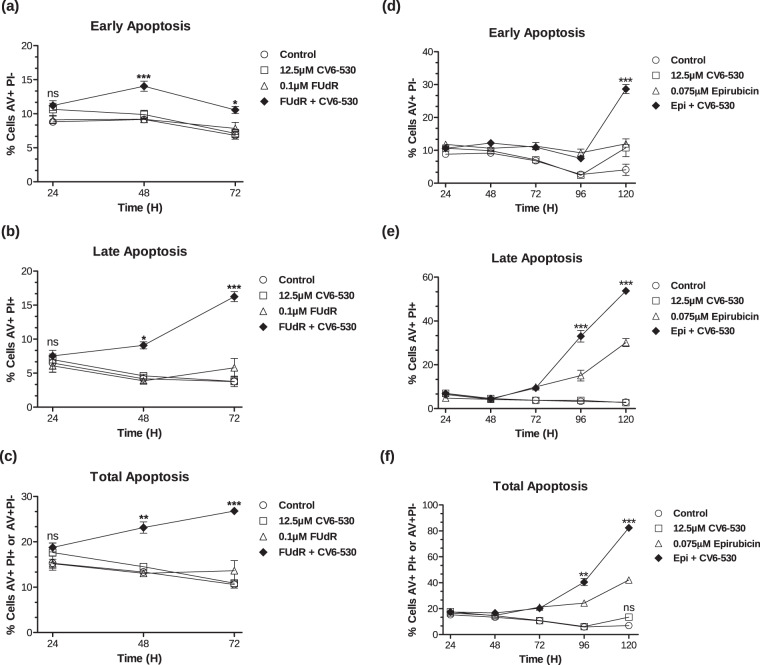
Inhibition of dUTPase enhances cell death induced by fluoropyrimidines and anthracyclines. MDA-MB-231 cells were treated with (**a–c**) 0.1 µM FUdR or 12.5 µM CV6-530 alone or in combination for 24 h when the media was replaced with drug-free media or (**d–f**) cells were treated with 0.075 µM epirubicin for 4 h or 12.5 µM CV6-530 alone or in combination for 24 h when the media was replaced with drug-free media. Cells were stained for Annexin V (AV) and propidium iodide (PI) and cell nuclei were stained with Hoechst. Cells were imaged on a high-content fluorescent microscope and quantified for AV+ and/or PI+ at 24, 48, 72, 96 and 120 h, as indicated. **a**, **d** Early apoptosis was quantified as cells stained as AV^+^/PI^−^. **b**, **e** Late apoptosis was quantified as cells stained as AV^+^/PI^+^. **c**, **f** Total apoptosis was quantified as cells stained AV^+^/PI^−^ and AV^+^/PI^+^. Data are presented as the mean ± SEM (*N* = 3, independent experiments). Statistical analysis determined by an unpaired, two-tailed Student *t*-test of treated relative to time-matched control. ns, not significant; *P < 0.05; **P < 0.01; ***P < 0.001.

The ability of the DUTi combinations to enhance apoptosis in the MDA-MB-231 cells was subsequently evaluated. DUTi alone did not induce a significant increase in apoptosis at any time point investigated. Low-dose 0.1 µM FUdR did not induce a significant increase in cell death compared to control, however, the combination of 0.1 µM FUdR with DUTi induced significant levels of total apoptosis at 48 h (23.1% ± 1.3, *P* = 0.0017) and 72 h (26.8% ± 0.5, *P* < 0.0001) compared to single-agent FUdR (Fig. 3A–C). 0.075 µM epirubicin alone induced significant increases in apoptosis after 96 h (24.3% ± 1.6, *P* = 0.0005) and 120 h (42.1% ± 2.2, *P* = 0.0003). The combination of epirubicin and DUTi induced a significant increase in apoptosis compared to epirubicin alone at both 96 h (40.6% ± 2.7, *P* = 0.0064) and 120 h (82.4% ± 1.1, *P* < 0.0001) (Fig. 3D–F).

**Fig. 4:**
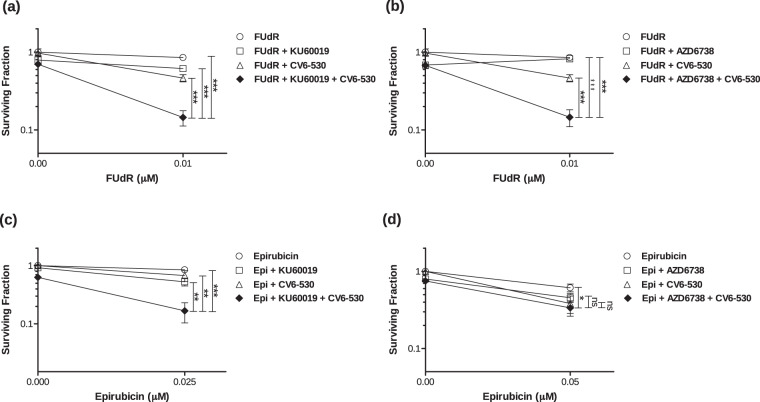
Inhibition of dUTPase leads to replication stress and persistent DNA double-strand breaks following combination treatment with chemotherapy. Cell survival was determined by colony formation assay. MDA-MB-231 cells were seeded into 24-well plates and treated with (**a**, **b**) 0.01 µM FUdR alone or in combination with 12.5 µM CV6-530 and/or (**a**) 2.5 µM ATM inhibitor (KU-60019) or (**b**) 100 nM ATR inhibitor (AZD6738) or (**c**, **d**) 0.025 or 0.05 µM epirubicin (4 h) alone or in combination with 12.5 µM CV6-530 (24 h) and/or (**c**) 2.5 µM ATM inhibitor (KU-60019, 24 h) or (**d**) 100 nM ATR inhibitor (AZD6738, 24 h). Drug-containing media was then replaced with drug-free media for 12–15 days and cells were allowed to form colonies (>50 cells). All data points are expressed as mean ± SEM (*N* = 3, independent experiments). ns, not significant; *P < 0.05; **P < 0.01; ***P < 0.001 by an unpaired, two-tailed Student *t*-test.

To distinguish between the different mechanisms of DNA damage being induced with DUTi combined with FUdR vs. epirubicin, specific inhibitors of both ATM (DSBs) and ATR (replication stalls and single-strand breaks (SSBs)) were used in combination with 0.01 µM FUdR or 0.05 µM epirubicin in combination with DUTi and the effect on cell survival following treatment was analyzed. The ATM inhibitor (ATMi; KU-60019) and ATR inhibitor (ATRi; AZD6738) potently inhibit their target and effectively sensitised glioma cells (ATMi) and NSCLC cells (ATRi) to radiation and were used at doses with confirmed target inhibition^18,19^. Inhibition of either ATM or ATR enhanced the effect of the combination of FUdR and DUTi on cancer cell lethality by 31.2% ± 6.1 (*P* = 0.0003) and 31.8% ± 6.1 (*P* = 0.0004), respectively (Fig. 4A, B). In contrast, only ATMi significantly increased cancer cell lethality with the combination of epirubicin and DUTi, with an observed increase in the lethality of 51.0% ± 13.9 (*P* = 0.0062) (Fig. 4C). Similar data were observed for ATMi in combination with doxorubicin and DUTi (Supplementary Fig. 6) These data would indicate that different types of DNA damage are being induced when DUTi is combined with different DNA-damaging chemotherapies. The FUdR and DUTi combination induces DNA damage which activates both ATM-dependent and ATR-dependent checkpoint signalling due to replication stalls and subsequent SSBs and DSBs, whereas epirubicin and DUTi results in DNA DSBs which activates primarily ATM-dependent checkpoint signalling with no apparent role for ATR. This is supported by the FUdR and DUTi combination causing both ɣH2AX signalling alone and co-localised ɣH2AX and 53BP1 suggesting a DNA damage profile consisting of stalled DNA replication, SSB and DSBs. Epirubicin in combination with DUTi only produced co-localised ɣH2AX and 53BP1 foci that persisted for longer than epirubicin alone, an indicator that the ability to repair these DNA DSBs was compromised (Supplementary Fig. 5).

### dUTPase inhibition promotes uracil misincorporation into DNA and drives the enhanced anti-cancer activity in combination with both fluoropyrimidine and anthracycline chemotherapies

**Fig. 5:**
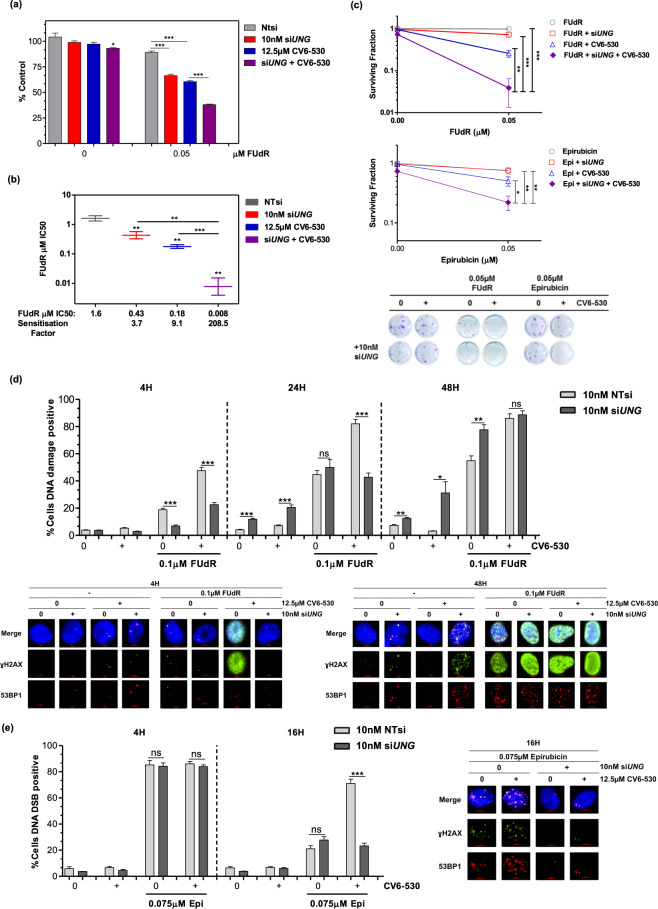
Uracil DNA glycosylase is required for the attempted repair of misincorporated uracil and the subsequent DNA damage induced by the base excision repair (BER) pathway. MDA-MB-231 cells were transfected with 10 nM NTsi or si*UNG* and seeded into (**a**, **b**) 96-well plates for growth inhibition assays or (**c**) 24-well plates for colony formation assays or (**d**, **e**) DNA damage immunofluorescence assays. **a**, **b** Growth inhibition was determined by CellTiter-Glo (Promega) following cells treated continuously for 96 h with 0.5 µM FUdR alone or in combination with dUTPase inhibition by 12.5 µM CV6-530. **a** Bar graph representing the growth inhibition results of 0.05 µM FUdR treated MDA-MB-231 cells with or without si*UNG* transfection and/or dUTPase inhibition by 12.5 µM CV6-530. Data are presented as the mean± SEM percentage growth compared with vehicle-treated control (*N* = 3, independent experiments). **b** Graph showing the IC_50_ values of FUdR in MDA-MB-231 cells treated with FUdR ± si*UNG* transfection and/or dUTPase inhibition by 12.5 µM CV6-530 calculated using Graphpad (Prism) and sensitisation factors were calculated by dividing the single-agent FUdR IC_50_ by the IC_50_ of the combination of FUdR and si*UNG* and/or 12.5 µM CV6-530. Data are presented as the mean ± SEM IC_50_. **c** Cell survival was determined by colony formation assay and representative images are shown to illustrate differences in colony formation capacity. Following transfection with NTsi or si*UNG*, MDA-MB-231 cells were treated with 0.05 µM epirubicin (4 h) or FUdR (24 h) alone or with 12.5 µM CV6-530 (24 h). Following treatment, media was replaced with drug-free media for 12–15 days and cells were allowed to form colonies (>50 cells). Data are presented as the mean ± SEM percentage colony formation compared with vehicle-treated control (*N* = 3, independent experiments). **d**, **e** Following transfection with NTsi or si*UNG* MDA-MB-231 cells in 24-well plates were treated with (**d**) 0.1 µM FUdR or (**e**) 0.075 µM epirubicin (Epi) alone or in combination with 12.5 µM CV6-530. Cells were fixed at the indicated time points. Cells were stained and imaged for ɣH2A.X (green) and 53BP1 (red). The line graph represents the mean ± SEM percentage cells positive for (**d**) DNA damage (ɣH2A.X) or (**e**) DNA double-strand breaks (>5 co-localised ɣH2A.X/53BP1 foci) quantified from *N* = 3 independent experiments, >100 cells were scored per experiment. Representative immunofluorescent images of ɣH2A.X and 53BP1 are shown on the right. All data points are expressed as mean ± SEM. ns, not significant; *P < 0.05; **P < 0.01; ***P < 0.001 by an unpaired, two-tailed Student *t*-test.

Misincorporated uracil in DNA is primarily repaired following recognition and excision by uracil-DNA glycosylase (UNG) which results in an apurinic/apyrimidininic site (AP) that is repaired by base excision repair (BER). We hypothesised that inhibition of dUTPase would lead to uracil misincorporation into DNA with subsequent induction of the DNA damage observed with both FUdR and anthracycline-based combinations. We silenced UNG by siRNA in MDA-MB-231 cells (Supplementary Fig. 7) and measured the effect on cell growth, survival and DNA damage following treatment with DUTi in combination with FUdR or epirubicin. Knockdown of UNG alone by si*UNG* had no impact on cell proliferation or survival but resulted in a small induction of DNA damage at 24 h and 48 h compared to non-targeting (NT) siRNA in MDA-MB-231 cells (Fig. 5A–D). As described previously, DUTi did not induce significant growth arrest, cell death or DNA damage, however, the combination of si*UNG* with DUTi induced a small reduction in growth (11.08% ± 3.9, *P* = 0.029) (Fig. 5A), survival (27.2% ± 8.8, *P* = 0.004) (Fig. 5C) and an increase in DNA damage at 24 h and 48 h (8.65% ± 1.8, *P* = 0.003 and 18.70% ± 8.3, *P* = 0.066, respectively) (Fig. 5D). Taken together, these results demonstrate that DUTi alone results in uracil misincorporation into DNA, which if not repaired by UNG leads to DNA damage and cell death.

Silencing of UNG sensitised MDA-MB-231 cells to FUdR, with significant increases in growth inhibition (Fig. 5A, B) and decreases in survival (Fig. 5C). When analysed for DNA damage, UNG silencing initially suppressed the DNA damage measured at 4 h following FUdR (*P* < 0.0001), however, by 48 h UNG-silencing induced a significant increase in DNA damage compared with NTsi (*P* = 0.0048) (Fig. 5D; Supplementary Fig. 8). As expected, UNG silencing had no impact on cell survival following single-agent epirubicin (Fig. 5C) and had no impact on the percentage of cells positive for DSBs induced at 4 h or 16 h (Fig. 5E). However, UNG-silencing in combination with DUTi significantly increased cancer cell lethality when used in combination with FUdR or epirubicin (Fig. 5C) and significantly increased growth inhibition with DUTi in combination with FUdR (Fig. 5A, B). These significant increases in DNA damage and cancer cell lethality when UNG is silenced in combination with dUTPase inhibition indicate a clear role for uracil misincorporation in the increased levels of DNA damage and lethality when used in combination with either FUdR or epirubicin.

**Fig. 6:**
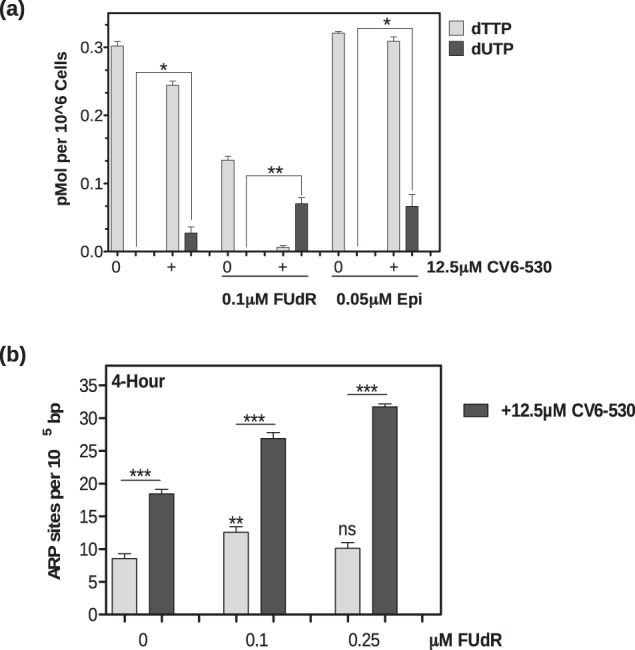
Inhibition of dUTPase enhances uracil pool expansion and uracil misincorporation in TNBC cells treated with FUdR or epirubicin. **a** MDA-MB-468 cells were treated for 24 h with 0.1 µM FUdR or 0.05 µM epirubicin (Epi) alone or in combination with 12.5 µM CV6-530 before cells were collected for nucleotide pool analysis using a previously described^31^ 96-well fluorescence-based assay to measure dTTP and dUTP levels. **b** MDA-MB-468 cells were treated with 0.1 or 0.25 µM FUdR alone or in combination with 12.5 µM CV6-530 before genomic DNA was collected using PureLink® Genomic DNA Mini Kit (Invitrogen) for ARP site analysis using a luminescence-based assay (Promega). All data points are expressed as mean ± SEM (*N* = 3 independent experiments). ns, not significant; *P < 0.05; **P < 0.01; ***P < 0.001 by an unpaired, two-tailed Student *t*-test.

We, therefore, examined the dUTP:dTTP nucleotide pool ratio by quantifying dTTP and dUTP in MDA-MB-468 cells. DUTi alone induced a small decrease in dTTP (*P* = 0.003) while simultaneously increasing low levels of dUTP (*P* = 0.014) (Fig. 6A) and a simultaneous increase in AP sites from dUTP misincorporation (*P* < 0.0001) (Fig. 6B). As expected, FUdR induced a > 50% decrease in dTTP, but no detectable increase in dUTP (Fig. 6A) and a significant, 46.9% increase in uracil misincorporation following 0.1 μM FUdR (*P* = 0.006) (Fig. 6B). The combination of DUTi and FUdR resulted in the largest decrease in dTTP of 0.296 pMol (±0.007, *P* < 0.0001) and the largest increase in dUTP pools of 0.140 pMol (±0.023, *P* = 0.004) (Fig. 6A) and the largest amount of uracil misincorporation into DNA (Fig. 6B). Similar results were observed in the MDA-MB-231 cells (Supplementary Fig. 7). Epirubicin alone induced no significant change in dTTP or dUTP levels. However, the combination of DUTi and epirubicin induced a significant 0.079pMol (±0.029, *P* = 0.027) increase in dUTP (Fig. 6A). This highlights the difference in mechanism between these combinations with FUdR and DUTi resulting in decreased dTTP pools and increased dUTP pools whereas epirubicin and DUTi resulting in no detectable change in dTTP. This is supported by exogenous thymidine addition to the media rescuing the effects of the FUdR combination, but not the epirubicin combination on cell survival (Supplementary Fig. 2).

### Inhibition of dUTPase sensitises TNBC cells to fluoropyrimidine and anthracycline chemotherapy in vivo

**Fig. 7:**
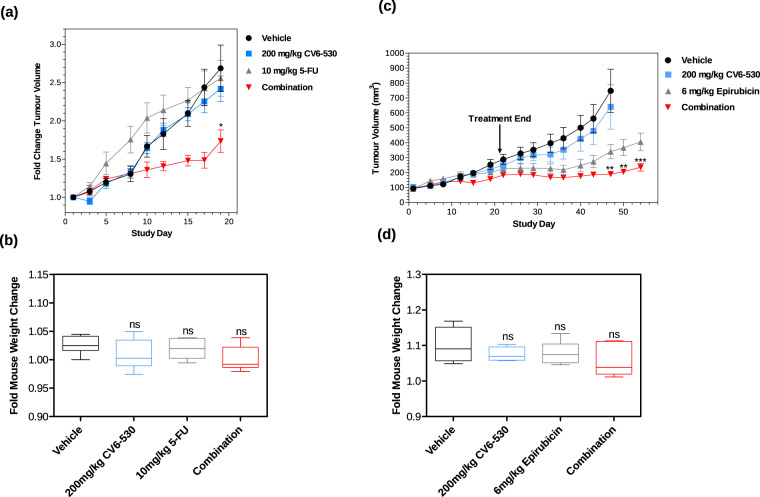
CV6-530 enhances the efficacy of SoC chemotherapies in TNBC in vivo xenograft model. **a** Line-graph representing the mean ± SEM tumour volume fold change of tumours implanted on mice treated with 200 mg/kg CV6-530 and/or 10 mg/kg 5-FU (*N* = 5/group). **b** Box-plot comparing the mean ± SEM fold change in body weights (day 19 compared with day 1) of mice treated with 10 mg/kg 5-FU and 200 mg/kg CV6-530 alone or in combination (*N* = 5/group). Centre line represents the median, bounds of the box represent upper and lower quartiles and whiskers represent the min and max values. **c** Line-graph representing the mean ± SEM tumour volume fold change of tumours implanted on mice treated with 200 mg/kg CV6-530 and/or 6 mg/kg epirubicin (*N* = 6/group). **d** Box-plot comparing the mean ± SEM fold change in body weights of mice treated with 6 mg/kg epirubicin and 200 mg/kg CV6-530 alone or in combination (*N* = 6/group). Centre line represents the median, bounds of the box represent upper and lower quartiles and whiskers represent the min and max values. One-way analysis of variance (ANOVA) for (**a**) and (**c**) and unpaired, two-tailed Student *t*-test for (**b**) and (**d**), *P < 0.05, **P < 0.005, ***P < 0.0001.

Having demonstrated conclusively in vitro the anti-cancer potency of the combinations of DUTi with chemotherapy, we investigated the potential efficacy in vivo using the MDA-MB-231 cells in a mouse xenograft model. Two studies were carried out to evaluate the efficacy and tolerability of DUTi with either 5-FU or epirubicin. MDA-MB-231 tumours treated with vehicle control showed a linear growth pattern over the course of both studies (Fig. 7). In both studies, DUTi (200 mg/kg CV6-530) resulted in no significant difference in tumour volumes (TV) compared to vehicle-treated controls (Fig. 7A, C). 5-FU at 10 mg/kg had no significant impact on TV compared to control (Fig. 7A). Epirubicin (6 mg/kg) induced a significant reduction in TV, which became increasingly apparent late in the study (D26) (Fig. 7C). Both combinations of 5-FU or epirubicin with DUTi significantly decreased TV compared to their respective single agents. DUTi enhanced the efficacy of 5-FU as observed after day 10, with a significant reduction in tumour volume on day 19 (*P* = 0.036). Similarly, DUTi enhanced the efficacy of epirubicin with a significant reduction in TV compared to single-agent from day 47 to the end of the study (*P* < 0.0001) (Fig. 7C). Importantly, there were no significant differences in mouse body weight (*P* > 0.05) or changes to mouse appearance or behavior across all treatment groups when compared to vehicle-treated mice suggesting these treatments were well-tolerated (Fig. 7B, D).

### dUTPase inhibition enhances the anti-cancer activity of the combination of 5-FU’s active metabolite, FUdR, plus epirubicin

**Fig. 8:**
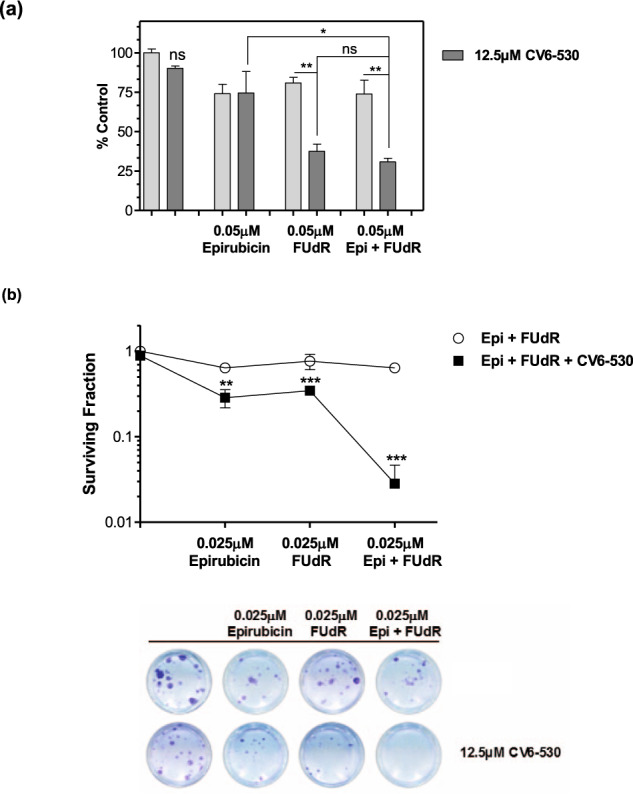
A potential effective triple combination for TNBC. **a** MDA-MB-231 cells were analysed for cell viability following dUTPase inhibition by 12.5 µM CV6-530 in combination with 0.05 µM FUdR and/or epirubicin (Epi) for 96 h and quantified with CellTiter-Glo assay (Promega). Bar graph representing the mean ± SEM percentage compared with vehicle-treated control (*N* = 3 independent experiments). **b** Cell survival was determined by colony formation assay following dUTPase inhibition by 12.5 µM CV6-530 (24 h) in combination with 0.025 µM FUdR (24 h) and/or epirubicin (4 h). Following treatment, media was replaced with drug-free media for 12–15 days and cells were allowed to form colonies (>50 cells). Representative images are shown to illustrate differences in colony formation capacity. All data points are expressed as mean ± SEM (*N* = 3 independent experiments). ns, not significant; *P < 0.05; **P < 0.01; ***P < 0.001 by an unpaired, two-tailed Student *t*-test.

Finally, to investigate the potential of utilising a DUTi in combination with both 5-FU’s active metabolite, FUdR, and epirubicin in a regimen similar to FEC (5-FU, epirubicin, cyclophosphamide), MDA-MB-231 cells were treated with both epirubicin and FUdR in combination with DUTi. While no significant increase in growth inhibition was observed with the triple combinations versus the combination of DUTi plus FUdR (Fig. 8A), the triple combination of FUdR, epirubicin and DUTi did significantly reduce survival compared to all other treatment conditions (Fig. 8B).

### Limited clinical utility of dUTPase as a predictive or prognostic marker for TNBC

To evaluate the predictive and prognostic value of *DUT* expression in breast cancer and TNBC, we have analysed the publicly available TCGA dataset. The analysis was carried out using the Breast Cancer Integrative Platform^20^. *DUT* expression was found to be significantly higher across all breast cancer subtypes in the analysis when compared to the adjacent normal tissue *(P* = 0.0005) (Supplementary Fig. 9A). We found no significant difference in *DUT* expression between TNBC and non-TNBC samples (*P* = 0.91) (Supplementary Fig. 9B) or between different pathological stages (*P* = 0.09) (Supplementary Fig. 9C). To investigate if *DUT* expression would correlate with proliferation, we performed a co-expression analysis of *DUT* and *MKI67* and found a significant positive correlation (Spearman correlation 0.07, *P* = 0.0193) (Supplementary Fig. 9D). Together, these results suggest that dUTPase expression may correlate with proliferation, which could explain the increased expression observed in breast cancer tumours compared to normal tissue.

To investigate the prognostic value of *DUT* in breast cancer, we examined the relationship between *DUT* expression and overall survival (OS). Analysis of all breast cancer subtypes together and using the median value of *DUT* expression, there was no significant prognostic value (*P* = 0.85) (Supplementary Fig. 9E).

To evaluate the predictive role of *DUT* in TNBC and to address the question if *DUT* expression could be predictive or prognostic in regards to TNBC patients receiving 5-FU/anthracycline-based regimens we carried out an analysis of breast cancer patients (*N* = 3955) with gene expression and survival analysis available on KM plotter^21,22^. Expression thresholds were set by the software selecting the cut-off with optimal performance. Expression of *DUT* correlated with relapse-free survival (RFS) (*P* = 0.0051) (Supplementary Fig. 10A), however, this did not translate to a significant difference in OS (*P* = 0.07) (Supplementary Fig. 10B) across all breast cancer patients, similar to the previous data set analysed (Supplementary Fig. 9). We next investigated *DUT* expression in the TNBC patients (*N* = 255) and found *DUT* expression had no significant impact on RFS (*P* = 0.21) (Supplementary Fig. 10C). We were then able to separate the TNBC patients who had received chemotherapy treatment and again investigate if *DUT* expression affected RFS; however, the total sample size was limited to 114 patients for this analysis. We found that in the TNBC chemotherapy-treated patients, *DUT* expression had no significant effect on RFS (*P* = 0.44) (Supplementary Fig. 10D). We could not analyse patients who specifically received 5-FU or anthracycline-containing regimens as this information was not available.

## Discussion

TNBC remains the most lethal BC subtype with poor response rates to the SoC chemotherapies and a lack of additional effective treatment options. Previous analysis by Brown et al. identified adaptive metabolic reprogramming of pyrimidine synthesis as an early event that promotes TNBC chemotherapy resistance^6^. In addition, work by Chen et al. demonstrated that tumours with concurrent low dUTPase activity and high ribonucleotide reductase activity would exhibit replication stress due to uracil misincorporation^23^. Building on this, we have demonstrated both in vitro and in vivo that inhibition of dUTPase, an enzyme that protects DNA from lethal uracil misincorporation, further sensitises TNBC cells to both fluoropyrimidines and anthracyclines, two distinct classes of chemotherapies that are components of SoC chemotherapy regimens. Previous publications have shown that inhibition of dUTPase sensitises cancer cells to TS-targeting chemotherapies^10–14,24,25^, however, this is the first paper demonstrating that directly targeting dUTPase can sensitise cancer cells to anthracyclines and simulated chemotherapy regimens containing both fluoropyrimidines and anthracyclines. This is of particular importance as TAS-114, a dual dUTPase and dihydropyrimidine dehydrogenase (DPD) inhibitor, is currently in early-phase clinical trials in combination with TS-targeting therapies (NCT02454062, NCT02855125, NCT02025803, NCT01610479) and an additional specific dUTPase inhibitor is in pre-clinical development^25^.

**Fig. 9:**
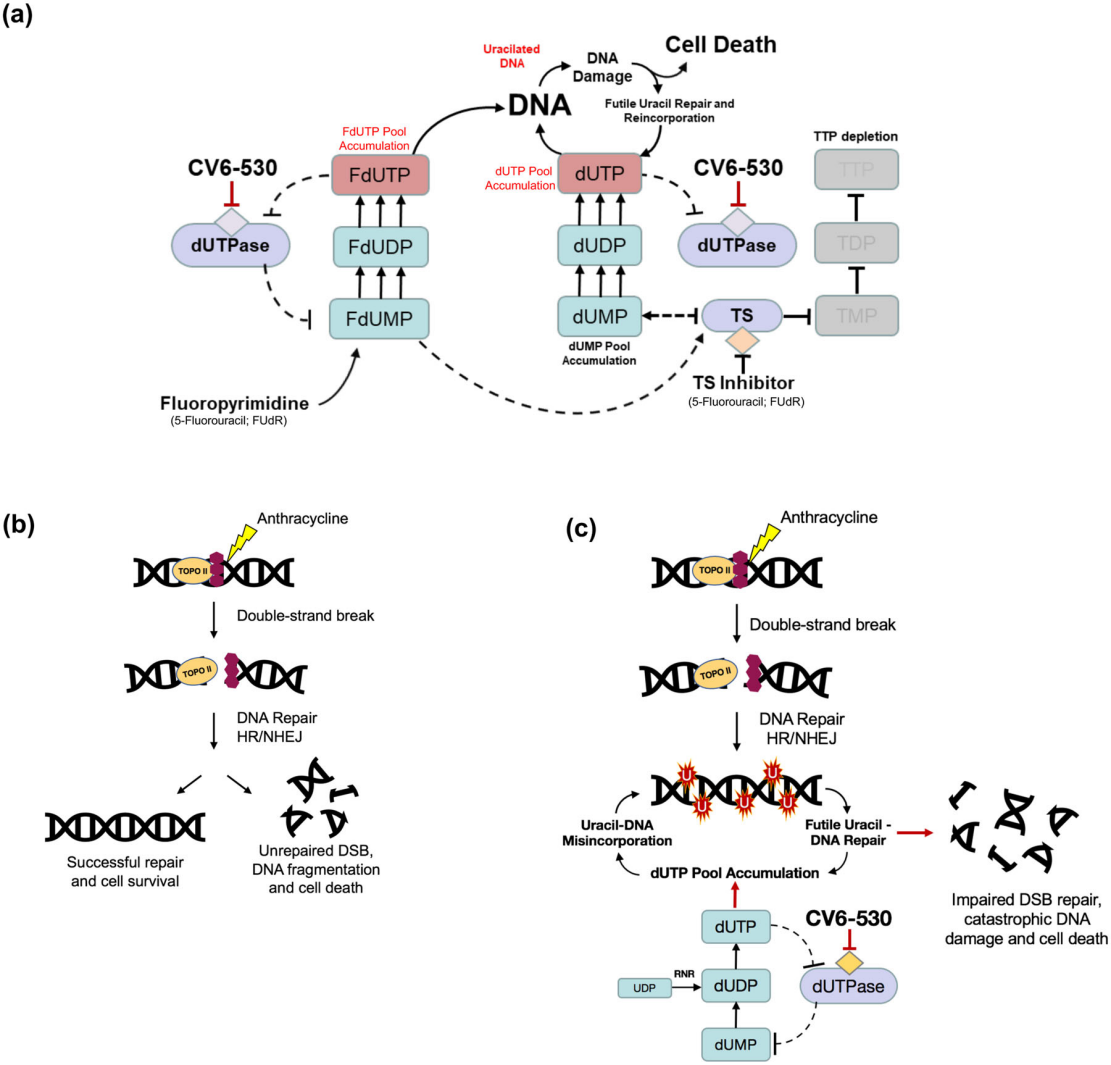
Schematic illustration of mechanisms influencing the combination of dUTPase inhibition with fluoropyrimidine and/or anthracyclines. **a** Schematic demonstrating the hypothesised consequences of combined dUTPase inhibition by CV6-530 and TS inhibition by fluoropyrimidine (e.g., 5-Fluorouracil (5-FU) or FUdR). Inhibition of dUTPase subsequently caused dUTP and FdUTP pool accumulation, while concurrent TS inhibition by FdUMP leads to TTP pool depletion. In combination, this leads to an increased (F)dUTP:TTP ratio and subsequent uracil misincorporation into DNA. Due to the persistent increase in dUTP:TTP pools there is a futile cycle of base excision repair (BER) leading to increased DNA damage and subsequently increased cell death. **b** Anthracyclines induce DNA double-strand breaks (DSBs) through inhibition of Topoisomerase 2 (Top2). The DNA DSBs are then repaired by non-homologous end-joining (NHEJ) or homologous repair (HR) leading to either cell survival or cell death depending on the success of the repair. **c** Anthracyclines induce DNA DSBs through inhibition of Top2. However, inhibition of dUTPase leads to dUTP pool accumulation following the phosphorylation of dUMP/dUDP. The resulting accumulation of dUTP pools leads to uracil misincorporation during the new DNA synthesis phase of DSB repair. Subsequent uracil-DNA repair by base excision repair (BER) compromised DSB repair leading to extensive DNA damage and increased cell death.

Anti-metabolites that target TS have been mainstay chemotherapies for >60 years^26^. We have shown in TNBC cell lines that DUTi synergizes in vitro and in vivo with fluoropyrimidines. The combination induced rapid depletion in dTTP pools and a corresponding increase in dUTP pools that resulted in the misincorporation of uracil during attempted DNA replication and repair, with significant increases in cancer cell lethality. The silencing of UNG, the key glycosylase responsible for initiating BER of misincorporated uracil, clearly enhanced the growth inhibition and lethality with DUTi in combination with FUdR further supporting the role of uracil misincorporation as the mechanisms driving the observed synergistic anticancer activity. Uracil misincorporation alongside TS inhibition is known to result in stalled DNA replication followed by collapsed replication forks. The subsequent attempts to repair DNA leads to repeated futile cycles of uracil excision and reincorporation, resulting in accumulating DNA damage and cancer cell lethality (Fig. 9A).

Anthracyclines induce DNA DSBs by inhibiting DNA topoisomerase 2 and the repair of DSBs requires the translocation of dNTP biosynthetic proteins to the sites of DNA damage and a subsequent localized increase in de novo nucleotide production for efficient DNA repair to occur^6,16^. The requirement for new DNA synthesis during DNA repair is dependent on the nature of the DNA damage and the repair pathway involved. The synthesis of new DNA appears critical for the efficient repair of anthracycline-induced DNA damage, but conversely, inhibition of dUTPase had no effect on the cytotoxicity of platinum agents, a class of chemotherapies typically associated with nucleotide excision repair. Previous research has suggested that the repair of DNA damage induced by anthracyclines is vulnerable to uracil misincorporation if dTTP biosynthesis is impaired^15,16^. We have demonstrated that inhibition of dUTPase, the gatekeeper enzyme which prevents dUTP accumulation, sensitises TNBC cells to anthracyclines in vitro and in vivo. Importantly, we demonstrate that dUTPase inhibition has no impact on the initial DNA damage induced by anthracyclines, an observation supported by no increase in growth inhibition with the combination, but rather the inhibition of dUTPase significantly impaired the ability of TNBC cells to resolve DNA DSBs. As DNA DSBs are among the most lethal types of DNA damage, the ability to prevent DSB repair in cancer cells represents an exciting strategy to improve the efficacy of anthracycline-based SoC chemotherapy. Our data strongly indicates that inhibition of dUTPase at the site of DNA DSB repair leads to aberrant uracil misincorporation into newly synthesized DNA and that this significantly undermines the repair process leading to the persistence of lethal unrepaired DNA damage (Fig. 9B). These contrasting mechanisms of synergy for DUTi in combination with fluoropyrimidines versus anthracyclines were apparent when the DNA damage response was impaired by inhibiting either ATM or ATR. Cancer cell lethality was significantly enhanced when ATM was inhibited in combination with DUTi and epirubicin, but not with ATR inhibition. In contrast, the combination of DUTi plus FUdR significantly enhanced cancer cell lethality with both ATM and ATR inhibition. This demonstrates the different types of DNA damage being induced with each chemotherapy in combination with DUTi. DUTi in combination with FUdR is typified by replication stress and the occurrence of DNA SSBs (signalled predominantly by ATR) and DSBs (signalled predominantly by ATM). DNA damage resulting from DUTi in combination with epirubicin however is mediated predominantly by the induction of DSBs (ATM signalling) and the inability to efficiently repair these DSBs due to uracil-misincorporation.

dUTPase inhibitors remain early in their clinical development. To date, there is limited clinical data in BC patients, with 23 BC patients enrolled onto a phase I study of TAS-114 in combination with capecitabine. Phase II trials in patients with advanced non-small-cell lung cancer (NSCLC) demonstrated that the combination of TAS-114 and S-1, an oral 5-FU derivative, resulted in an improved response rate without benefit in progression-free survival compared to S-1 alone^27^. However, this limited improvement was in heavily pre-treated patients that had been previously treated with ≥2 previous chemotherapy regimens. In addition, TAS-114 also inhibits the enzyme DPD which is required for the body to break down 5-FU. This dual inhibition results in potential dosage limitations when used in combination with fluoropyrimidines, as demonstrated by a dose-escalation study of TAS-114 in combination with capecitabine concluding that a 30% standard dose of capecitabine resulted in equivalent 5-FU exposure as 100% capecitabine alone (NCT02025803). A specific dUTPase inhibitor may have a larger therapeutic window when combined with fluoropyrimidines compared to a dual dUTPase and DPD inhibitor. In addition, these clinical trials were not biomarker-driven. Our results suggest that while dUTPase expression may correlate with breast cancer tumour proliferation, it is not associated with clinical outcome to chemotherapy and at present appears to have limited clinical utility as a biomarker for breast cancer. However, ongoing studies in the group are investigating alternative biomarkers within the nucleotide biosynthesis and DNA damage repair pathways that may serve as biomarkers in these settings. These data encourage a future biomarker-driven trial in the various subtypes of BC, but particularly TNBC as this subtype has only been marginally touched by personalized medicine approaches to date^28^.

While evidence of the potential benefits of the combination of a fluoropyrimidine and a specific dUTPase inhibitor would first come from early phase trials in heavily pre-treated metastatic breast cancer (e.g., through a breast cancer-specific phase Ib expansion cohort), use in the earlier disease setting may provide further benefit. Investigation of the combination in the neoadjuvant setting would allow sequential tumour-based and circulating biomarkers to be assessed, and possible predictive biomarkers developed. In the adjuvant setting, the potential utility of sequential adjuvant capecitabine after standard chemotherapy regimens has been explored, and the SYSUCC-001 trial recently demonstrated significant benefit with acceptable toxicities for women with stage II/III TNBC^29^. The addition of a dUTPase inhibitor to a fluoropyrimidine in this adjuvant setting in this poor prognosis subtype has the potential to further reduce the rate of disease recurrence and improve overall survival.

Our results demonstrate conclusively that inhibition of dUTPase enhances the in vitro and in vivo efficacy when used in combination with both fluoropyrimidines and anthracyclines in TNBC models. More importantly, we explored the triple combination of a dUTPase inhibitor in combination with two classes of agents that are frequently used in combination in TNBC and noted striking anticancer activity. These observations provide the rationale for the evaluation of this triple combination as a promising therapeutic strategy in TNBC. The importance of this discovery is pertinent as inhibitors of dUTPase are currently in early phase clinical evaluation and thus identifying the most effective clinical application for these new agents is of utmost importance^24,27,30^.

## Methods

### Cell culture

All cell lines were obtained from ATCC. MDA-MB-231 cells were maintained in RPMI (Sigma). MDA-MB-468 and CAL51 cells were maintained in DMEM and DMEM:F12, respectively (Sigma and Gibco/Invitrogen). Media contained 10% FBS (Sigma), 2mM L-glutamine (Sigma), 1 mM Sodium Pyruvate (Sigma) and 50 μg/mL Pen-strep (Sigma). Passage numbers were recorded and cells were maintained in culture for < passage 15 and were checked routinely for mycoplasma (MycoAlert™ Mycoplasma Detection Kit, Lonza).

### Chemotherapy agents and inhibitors

Doxorubicin (Sigma-Aldrich), epirubicin, AZD6738 and KU60019 (Selleck Chemicals) were dissolved in DMSO (Sigma). 5-fluorodeoxyuridine (FUdR; Sigma) and cisplatin (Abcam) were dissolved in sterile cell culture water (Sigma). Carboplatin (Hospiara, UK) was supplied at 10 mg/mL. CV6-530, a small molecule tool compound shown to inhibit dUTPase, was supplied by CV6 Therapeutics in DMSO. All drugs were sterile filtered and aliquoted to avoid freeze-thaw cycles. For in vivo studies, compounds were purchased from Sigma-Aldrich.

### Antibodies

For immunofluorescence imaging antibodies targeting pH2A.X^S139^ (1:5000, Millipore), 53BP1 (1:3000, Novus Biologicals), goat anti-mouse IgG-HRP (1:2000, Santa Cruz) and goat anti-rabbit IgG-AP (1:2000, Santa Cruz) were utilized. For immunoblots, dUTPase (1:500, Sigma-Aldrich), β-Actin (1:5000, Sigma-Aldrich), anti-mouse (1:2000, Cell Signalling Technology) and anti-rabbit (1:2000, Cell Signalling Technology) were utilized.

### Colony formation

Long-term cell survival was assessed by colony formation assay. Briefly, cells were seeded (150–500 cells/well) in 24-well plates. Cells were treated the following day for 24 h and then the media was replaced with drug-free media and cells were allowed to grow for 10–14 days. Colonies were fixed with ice-cold 70% methanol and stained with crystal violet. Colonies (>50 cells) were counted using GelCount^TM^ (Oxford Optronix) and expressed as percentage survival compared to untreated controls.

### Growth inhibition

Cells were seeded in white 96-well plates (Costar®) and left overnight to attach. Cells were then exposed to indicated concentrations of drugs and quantified by using CellTiter-Glo (Promega) according to the manufacturer’s guidelines and expressed as percentage growth compared to untreated controls.

### RNA interference

For siRNA silencing, SMARTpool of 4 siRNA sequences (Dharmacon) were used (Supplementary Table 1). Forward transfections were carried out using Lipofectamine RNAiMax transfection reagent (Life Technologies) according to the manufacturer’s guidelines. Validation of knockdown was verified by mRNA and protein expression.

### Quantitative real-time PCR (qRT-PCR)

RNA was isolated using RNeasy mRNA isolation kit (Qiagen) according to the manufacturer’s guidelines. mRNA was quantified using the CLARIOstar (BMG Labtech) plate reader and 50 ng was reverse transcribed to cDNA using the Sensiscript Reverse Transcription Kit (Qiagen). qRT-PCR was carried out on the cDNA using a LightCycler® 480 II (Roche) with fluorescent primer-probe sets (Supplementary Table 2). *DUT* and *UNG* were normalised to *ACTB* and quantified using ΔΔCT method.

### Immunoblotting

For immunoblotting analysis, the protein was isolated from cells by lysis in radioimmunoprecipitation buffer (Sigma) supplemented with protease inhibitor (1:500) (Calbiochem®), 0.01 mM sodium fluoride, and 1 mM sodium orthovanadate. Cell debris was cleared by centrifugation and protein concentration was measured using the Pierce BCA Protein Assay (Thermo Scientific) according to the manufacturer’s guidelines. Protein was diluted in 2× Laemmli Sample Buffer (Sigma) before being loaded into acrylamide gels and resolved by SDS-PAGE. Resolved protein was then transferred to a PVDF membrane (GE Healthcare, Life Sciences). Blots were blocked in 5% non-fat dry milk in 0.1% PBS-Tween (PBS-T) (Sigma-Aldrich) before being incubated with primary antibody at 4 ^o^C overnight. The membrane was then washed 3× in PBS-T before incubation with an HRP-conjugated secondary antibody for 1 h at room temperature (RT) on a rocker. The membrane was then washed again 3× in PBS-T before signal development using HRP substrate chemiluminescence (Luminata™, Merck) and imaged on the G:BOX Chemi XX6 (Syngene). All blots derived from the same experiment were processed in parallel.

### Immunofluorescence

Cells plated and treated on coverslips (VWR) were fixed with 4% formaldehyde on ice for 10 min, washed with PBS, permeabilised with 0.4% Triton® X-100 (Sigma) in PBS at RT for 20 min, and then blocked with 3% BSA in PBS for 30 min at RT before incubation with the primary antibody in 3% BSA for 1 h at RT. Coverslips were then washed 3× with PBS before incubation with secondary antibodies then washed and then mounted onto microscope slides (VWR) using ProLong^TM^ Gold containing DAPI (Invitrogen). The cells were imaged using a Nikon Fluorescent Microscope with filters for DAPI, TexasRed, and Fluorescein isothiocyanate (FITC) and subsequently analysed using NIS elements 4.20 and Image J software.

### Apoptosis and cell death assay

Apoptosis and cell death were assessed by imaging of Annexin V (AV)/Propidium Iodide (PI). Briefly, cells were seeded into black glass-bottom 96-well plates (Cellvis, P96-1.5H-N) and treated the following day. Cells were stained for AV and PI, with cell nuclei stained with Hoechst. Cells were imaged on a Thermo Scientific ArrayScanXTI and quantified for AV+ and/or PI+ cells.

### ARP site assay

Genomic DNA was isolated following treatment using PureLink^®^ Genomic DNA Mini Kit (Invitrogen) according to the manufacturer’s guidelines. Apurinic/apyrimidinic (AP) sites were measured using a luminescence-based assay with an aldehyde reactive probe (ARP) which binds to AP sites (Promega). AP sites are formed following uracil misincorporation and subsequent excision by UNG. To assess the amount of uracil misincorporation the assay was combined with UNG silencing (si*UNG)*, to allow misincorporated uracil to accumulate, and subsequently incubating the genomic DNA with recombinant UNG (New England Biolabs) to induce AP site formation.

### Nucleotide pool assay

Treated cells were harvested and nucleotides were isolated by resuspending cell pellets in 500 µL of ice-cold 60% methanol, vortexed vigorously to resuspend before sonication (Qsonica). Samples were then centrifuged to remove cell debris and then the supernatants were passed through 3 K centrifugal filters to remove macromolecules (Nanosep, PALL, Life Sciences). The filtrate was evaporated under a centrifugal vacuum and the resultant pellet resuspended in DUT buffer (50 mM Tris-HCl, 50 mM NaCl, 10 mM MgCl_2_). dTTP and dUTP levels were quantified as previously described^31^, using a 96-well fluorescence-based assay.

### MDA-MB-231 xenograft studies

All procedures were performed in accordance with the guidelines provided by the Animals (Scientific Procedures) Act 1986 (ASPA), and subsequent amendments. Healthy female Balb/c mice (4–6 weeks old) weighing between 17–20 g were purchased from Envigo. Prior to implantation, cells were screened for mycoplasma, confirmed to be negative, and were further inspected microscopically before being harvested, counted and suspended in PBS. Xenografts were established by the injection of 6 × 10^6^ cells in 100 µL PBS:Matrigel (1:1; Corning) using a 27-gauge sterile needle (Becton Dickinson). Once palpable, tumours were measured every 2 days using digital calipers and tumour volume (TV) was calculated using the modified Ellipsoid equation: 1/2(Length × Width^2^). Mouse body weight was measured every 2 days using digital scales as a general indicator of toxicity and/or general physical condition. Tumours were not allowed to exceed a volume of 1000 mm^3^. At TV’s of 100 mm^3^, mice were randomized into control and treatment groups of 5-FU (10 mg/kg), epirubicin (6 mg/kg) and CV6-530 (200 mg/kg) and combinations of CV6-530 with 5-FU or epirubicin. CV6-530 was administered daily on days 1–19 by oral gavage. 5-FU was administered every other day by intraperitoneal (i.p.) injection. Epirubicin was administered on days 1, 8, 15 by intravenous (i.v.) injection.

### Statistical analysis

Unless otherwise stated, experiments were performed in triplicate and results expressed as the mean ± SEM. Data were analysed using a two-tailed unpaired *t*-test or ANOVA using Graphpad (Prism), where *P* < 0.05 was considered statistically significant.

### Reporting summary

Further information on research design is available in the Nature Research Reporting Summary linked to this article.

## Supplementary information

Supplementary Information

Reporting Summary Checklist

## Data Availability

The data generated and analysed during this study are described in the following figshare data record: 10.6084/m9.figshare.14045951^32^. All the data underlying Figs. 1–8 of the related article, including cell survival, growth inhibition, DNA damage analysis, apoptosis, nucleotide pool and in vivo data, along with data underlying Supplementary Figs. 1, 2, 3, 4, 5, 7, 8 and Western Blot images for Supplementary Fig. 1a, b, are openly available as part of the figshare data record.
