# Supplementary material for: Targeting nucleotide metabolism enhances the efficacy of anthracyclines and anti-metabolites in triple-negative breast cancer
Source: NPJ Breast Cancer. 2021 Apr 6;7:38. doi: 10.1038/s41523-021-00245-5 (PMC8024381; doi:10.1038/s41523-021-00245-5)
Supplement: Supplementary file 1 — Supplementary Information [file 41523_2021_245_MOESM1_ESM.pdf]

Targeting nucleotide metabolism enhances the efficacy of anthracyclines and anti-metabolites in triple-negative breast cancer.

Craig Davison[1], Roisin Morelli[1], Catherine Knowlson[1], Melanie McKechnie[1], Robbie Carson[1], Xanthi Stachea[1], Kylie A McLaughlin[3], Vivien E Prise[3], Kienan Savage[1], Richard H Wilson[2], Karl A Mulligan[3], Peter M Wilson[3], Robert D Ladner[1†] and Melissa J LaBonte[1†*]

[1] Medicine, Dentistry and Biomedical Sciences: Patrick G Johnston Centre for Cancer Research, Queen's University Belfast, Belfast, BT9 7AE, UK
[2] Translational Research Centre, University of Glasgow, Glasgow, G12 8QQ, UK
[3] CV6 Therapeutics (NI) Ltd, Belfast, BT9 7AE, UK

*To whom correspondence should be addressed. Tel: +44 2890972789; Email: m.labontewilson@qub.ac.uk
† The authors wish it to be known that, in their opinion, the last two authors should be regarded as Joint Senior Authors

TABLE OF CONTENTS

Supplementary Figure 1. Inhibition of dUTPase does not alter TNBC survival.

Supplementary Figure 2. Thymidine addition prevents cell death following TS inhibition by FUdR in TNBC.

Supplementary Figure 3. Inhibition of dUTPase sensitises MDA-MB-231 cells to doxorubicin but not cisplatin or carboplatin.

Supplementary Figure 4. Imbalanced nucleotide pools following dUTPase inhibition in combination with Epirubicin induced persistent DNA DSBs.

Supplementary Figure 5: Imbalanced nucleotide pools following dUTPase inhibition in combination with FUdR induced rapid and robust DNA damage.

Supplementary Figure 6. DNA damage induced following combination treatments incorporating dUTPase inhibition require Uracil DNA glycosylase (UDG) for the recognition and attempted repair of misincorporated uracil.

Supplementary Figure 7: dUTPase inhibition results in persistent DNA double-strand breaks following doxorubicin treatment in MDA-MB-231 cells.

Supplementary Figure 8. Inhibition of dUTPase enhances uracil pool expansion and uracil misincorporation in MDA-MB-231 cells treated with FUdR.

Supplementary Figure 9. dUTPase expression does not correlate with overall survival for breast cancer patients from the TCGA data.

Supplementary Figure 10. Expression of dUTPase does not correlate with survival in breast cancer patients or triple negative breast cancer (TNBC) subtype.

Supplementary Table 1: Experimental siRNA sequences.

Supplementary Table 2: qRT-PCR primer-probe sets.

SUPPLEMENTARY FIGURE 1

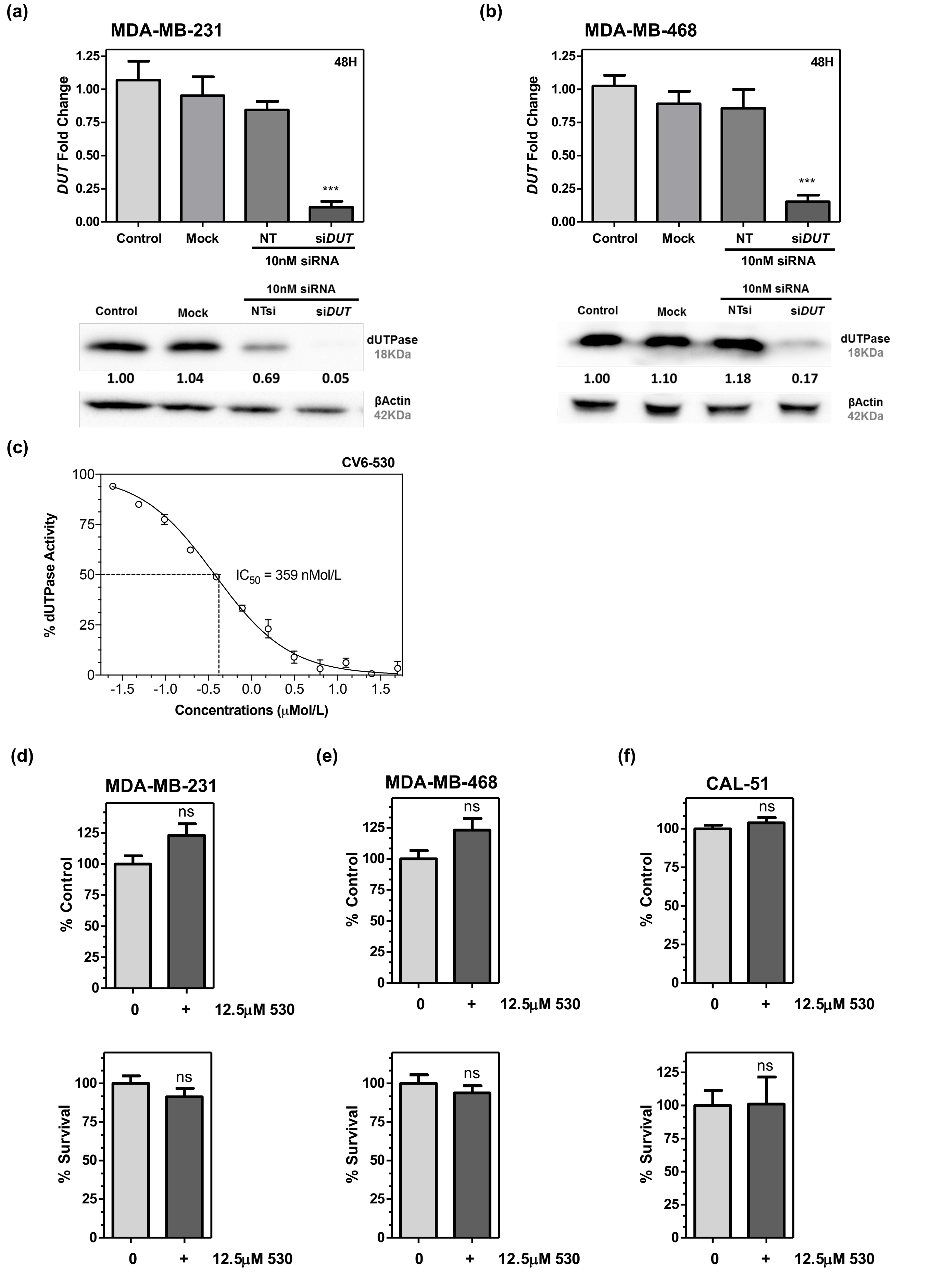

Supplementary Figure 1. Inhibition of dUTPase does not alter TNBC survival. (a-b) MDA-MB-231 and MDA-MB-468 cells were transfected with 10nM NTsi or si*DUT* and knockdown of dUTPase was demonstrated by qRT-PCR at 48H and Western blot 72H post-transfection. **(c)** dUTPase enzymatic activity was measured following increasing doses of CV6-530. Enzymatic inhibition was calculated at the IC_{50}. **(d-f)** MDA-MB-231, MDA-MB-468 and CAL-51 cells were seeded into 96-well plates for growth inhibition assay and 24-well plates for colony formation assay and treated with 12.5μM CV6-530. All data points are expressed as mean±SEM (N=3). ns, not significant; ***, $P<0.001$ by an unpaired, two-tailed Student *t*-test.

SUPPLEMENTARY FIGURE 2

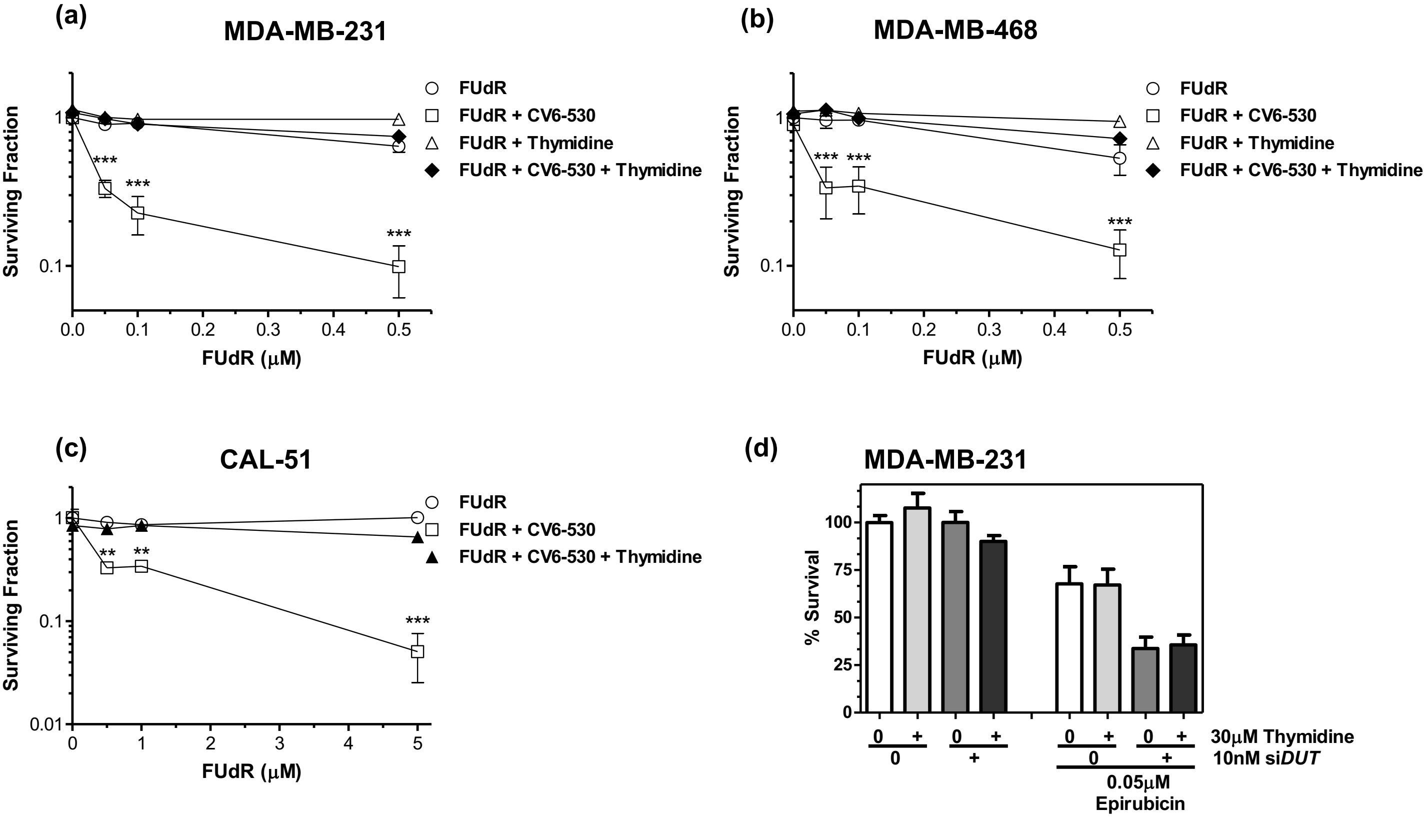

Supplementary Figure 2. Thymidine addition prevents cell death following TS inhibition by FUdR in TNBC. (a-c) Cell survival was determined by colony formation assay in MDA-MB-231, MDA-MB-468 and CAL-51 cells, where cells were treated with FUdR at the indicated doses plus 12.5μM CV6-530 and 30μM thymidine. Cells were allowed to form colonies (>50 cells) for 10-12 days. **(d)** Following NTsi or 10nM si*DUT* transfection MDA-MB-231 cells were reseeded into 24-well plates for colony formation assay to determine cell survival. MDA-MB-231 cells were treated with 0.05μM Epirubicin for 4H alone and in combination with 30μM thymidine. Cells were allowed to form colonies (>50 cells) for 10-12 days. All data points are expressed as mean±SEM (N=3). ns, not significant; **, *P*<0.01; ***, *P*<0.001 by an unpaired, two-tailed Student *t*-test.

SUPPLEMENTARY FIGURE 3

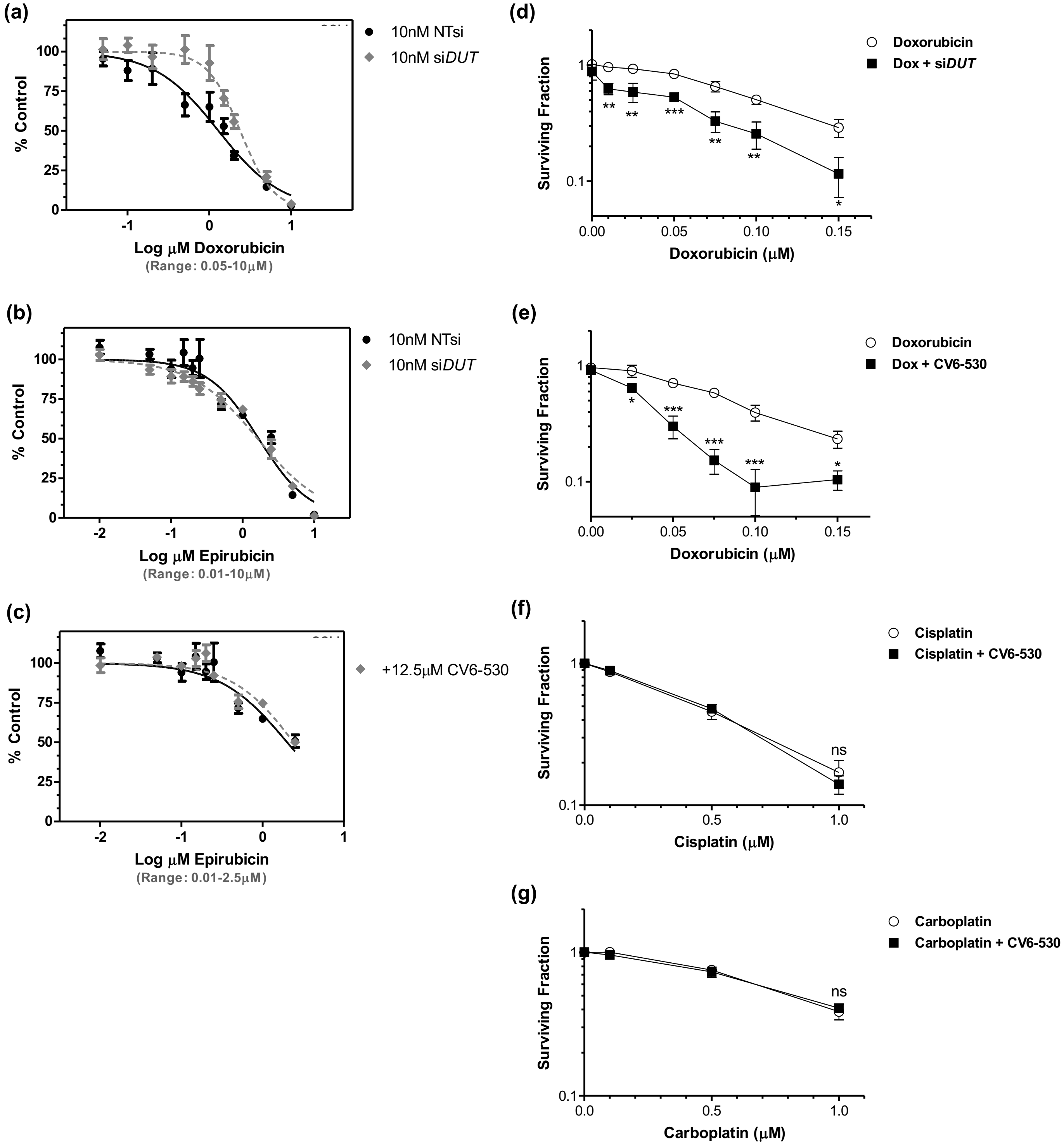

Supplementary Figure 3. Inhibition of dUTPase sensitises MDA-MB-231 cells to doxorubicin but not cisplatin or carboplatin. (a-b) Following 10nM si*DUT* or NTsi transfection MDA-MB-231 cells were reseeded into 96-well plates for growth inhibition assay. MDA-MB-231 cells were analysed for cell viability by Cell Titer Glo (Promega) following treatment with 0.01-10 μM doxorubicin or epirubicin at 96H. **(C)** MDA-MB-231 cells were seeded into 96-well plates for growth inhibition assay. MDA-MB-231 cells were analysed for cell viability by Cell Titer Glo (Promega) following treatment with 0.01-2.5 μM epirubicin alone or in combination with 12.5 μM CV6-530 at 96H. **(d)** Following 10nM si*DUT* or NTsi transfection MDA-MB-231 cells were reseeded into 24-well plates for colony formation assay to determine cell survival. MDA-MB-231 cells were treated 0.01-0.15 μM Doxorubicin for 4H. Cells were allowed to form colonies (>50 cells) for 10-12 days. **(e-g)** Cell survival was determined by colony formation assay, where cells were treated (**e**) 0.025-0.15 μM Doxorubicin (4H), (**f**) 0.1-1.0 μM Cisplatin (24H), or (**g**) 0.1-1.0 μM Carboplatin (24H) alone or in combination with 12.5μM CV6-530 (24H). Cells were allowed to form colonies (>50 cells) for 10-12 days. All data points are expressed as mean±SEM (N=3). ns, not significant; *, $P<0.05$; **, $P<0.01$; ***, $P<0.001$ by an unpaired, two-tailed Student *t*-test.

SUPPLEMENTARY FIGURE 4

(a)

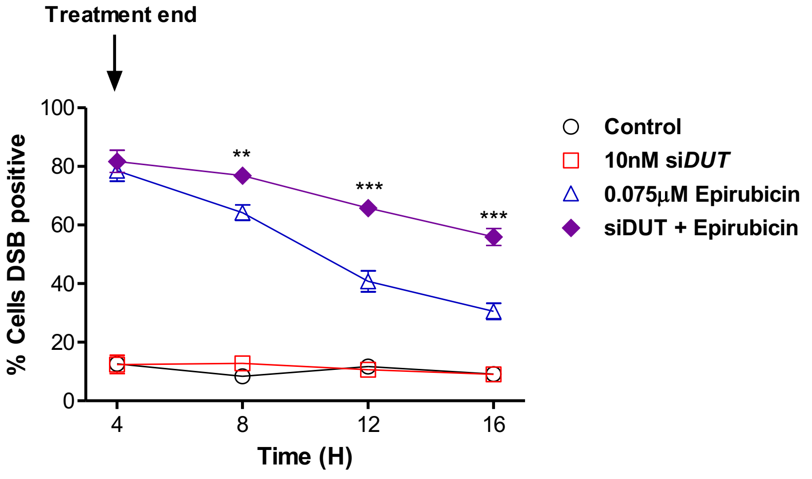

(b)

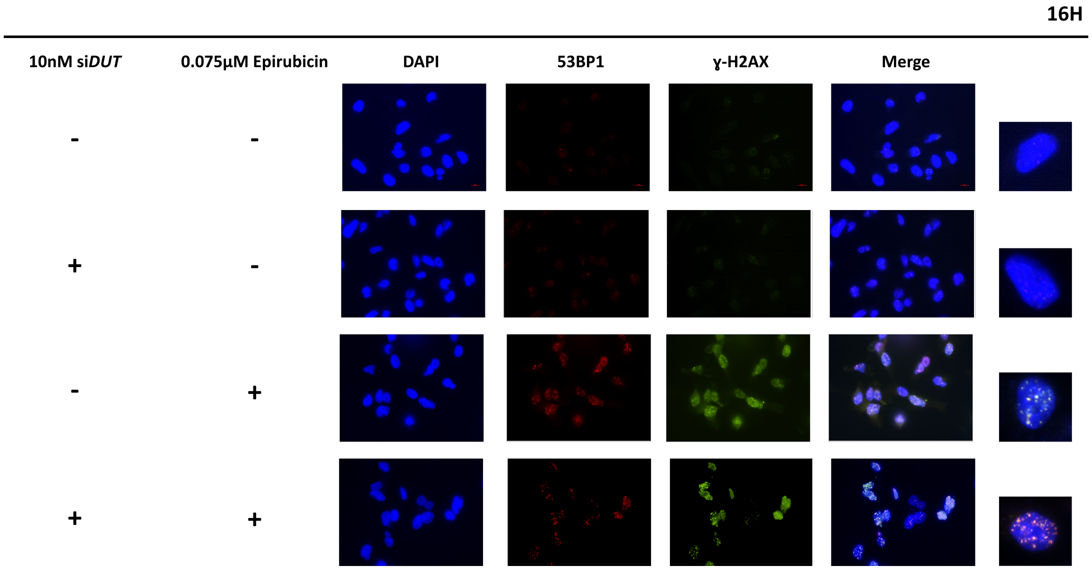

Supplementary Figure 4. Imbalanced nucleotide pools following dUTPase inhibition in combination with epirubicin induced persistent DNA DSBs. Following transfection with 10nM NTsi or si*DUT* MDA-MB-231 cells were reseeded onto coverslips in 24-well plates and were treated with 0.075µM Epirubicin for 4H prior to the media being replaced with drug-free media. Cells were fixed at indicated time points before being stained and imaged for ɣH2AX (green) and 53BP1 (red) foci by immunofluorescence. **(a)** Line graphs represent the mean±SEM percentage cells positive for DNA damage or DNA double-strand breaks (>5 ɣH2AX and 53BP1 co-localised foci/cell). **(b)** Representative images are shown with a zoomed in merged images of single cells shown on the far right. All data points are expressed as mean±SEM (N=3). ns, not significant; **, *P*<0.01; ***, *P*<0.001 by an unpaired, two-tailed Student *t*-test.

SUPPLEMENTARY FIGURE 5

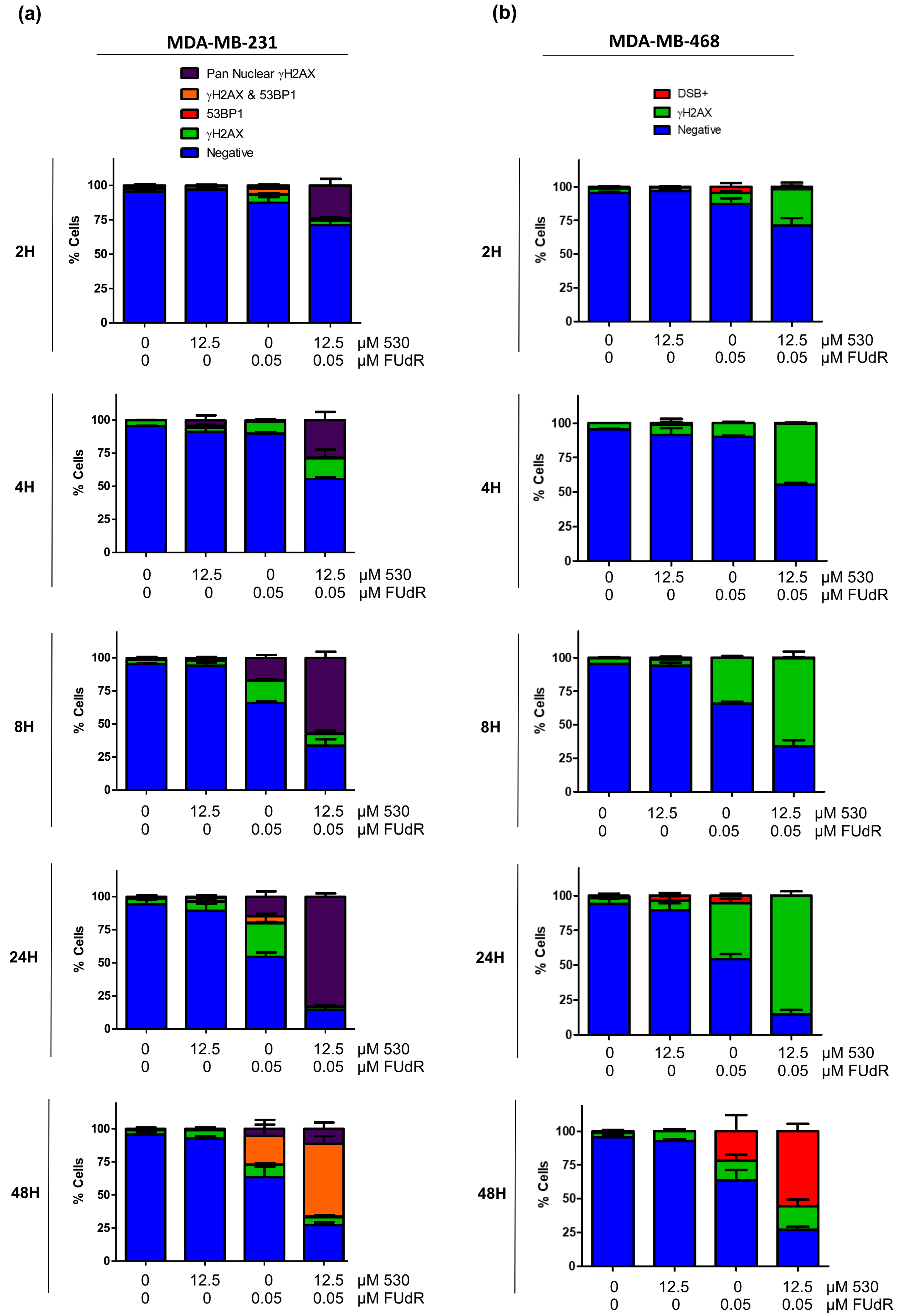

Supplementary Figure 5: Imbalanced nucleotide pools following dUTPase inhibition in combination with FUdR induced rapid and robust DNA damage. (a) MDA-MB-231 and **(b)** MDA-MB-468 cells seeded onto coverslips in 24-well plates and were treated with 0.05µM FUdR alone or in combination with 12.5µM CV6-530 for 24H prior to the media being replaced with drug-free media. Cells were fixed at indicated time points (2H-48H) before being stained and imaged for ɣH2AX and 53BP1 foci by immunofluorescence. Bar graphs represent the mean±SEM percentage cells positive for DNA damage as measured by ɣH2AX or DNA double-strand breaks (>5 ɣH2AX and 53BP1 co-localised foci/cell). All data points are mean±SEM (N=3).

SUPPLEMENTARY FIGURE 6

(a)

24H

siUNG	12.5μM 530	0.1μM FUdR	DAPI	53BP1	γ-H2AX	Merge
-	-	-				
+	-	-				
-	+	-				
+	+	-				
-	-	+				
+	-	+				
-	+	+				
+	+	+				

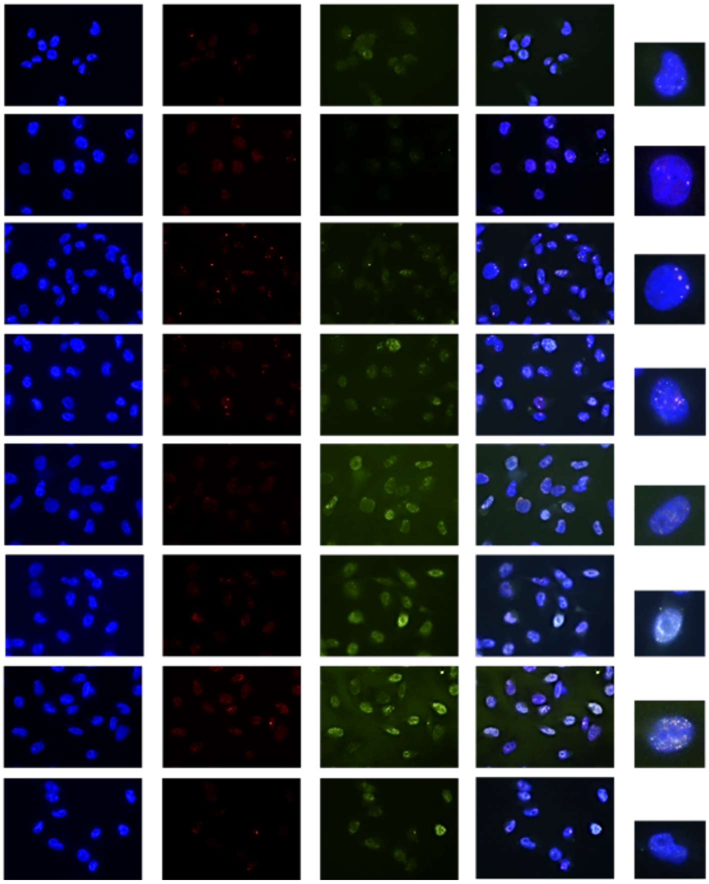

(b)

48H

siUNG	12.5μM 530	0.1μM FUdR	DAPI	53BP1	γ-H2AX	Merge
-	-	-				
+	-	-				
-	+	-				
+	+	-				
-	-	+				
+	-	+				
-	+	+				
+	+	+				

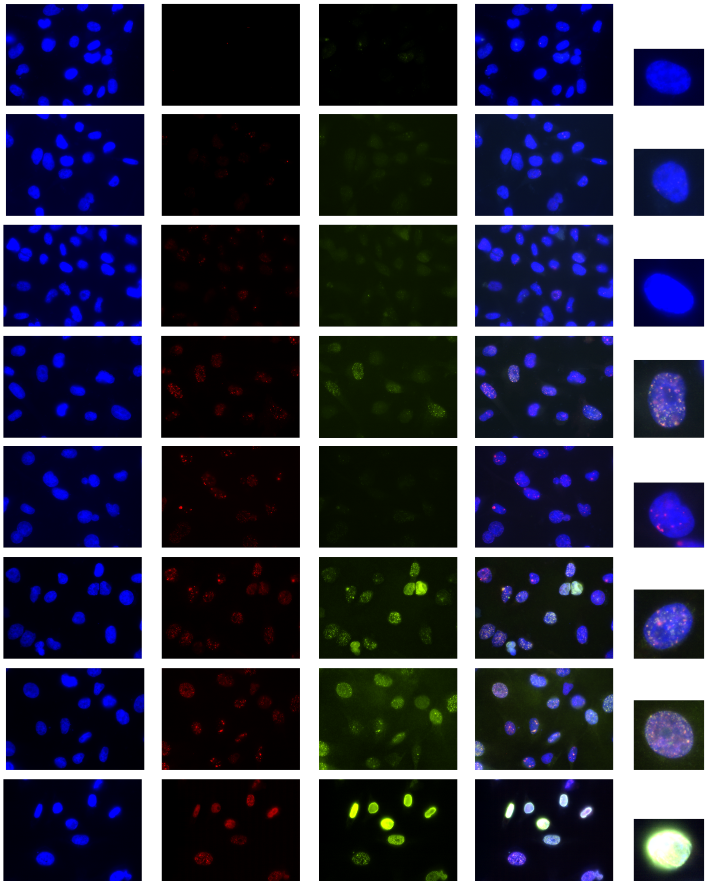

Supplementary Figure 6. DNA damage induced following combination treatments incorporating dUTPase inhibition require Uracil DNA glycosylase (UDG) for the recognition and attempted repair of misincorporated uracil. MDA-MB-231 cells were transfected with 10nM NTsi or si*UNG* for 24H prior to being reseeded into 24-well plates for DNA damage immunofluorescence assays. MDA-MB-231 were treated with 0.1μM FUdR alone or in combination with 12.5μM CV6-530. Cells were fixed on cover slips at **(a)** 24H and (**b**) 48H and stained and imaged for ɣH2AX (green) and 53BP1 (red). Representative images are shown with a zoomed in merged images of single cells shown on the far right.

SUPPLEMENTARY FIGURE 7

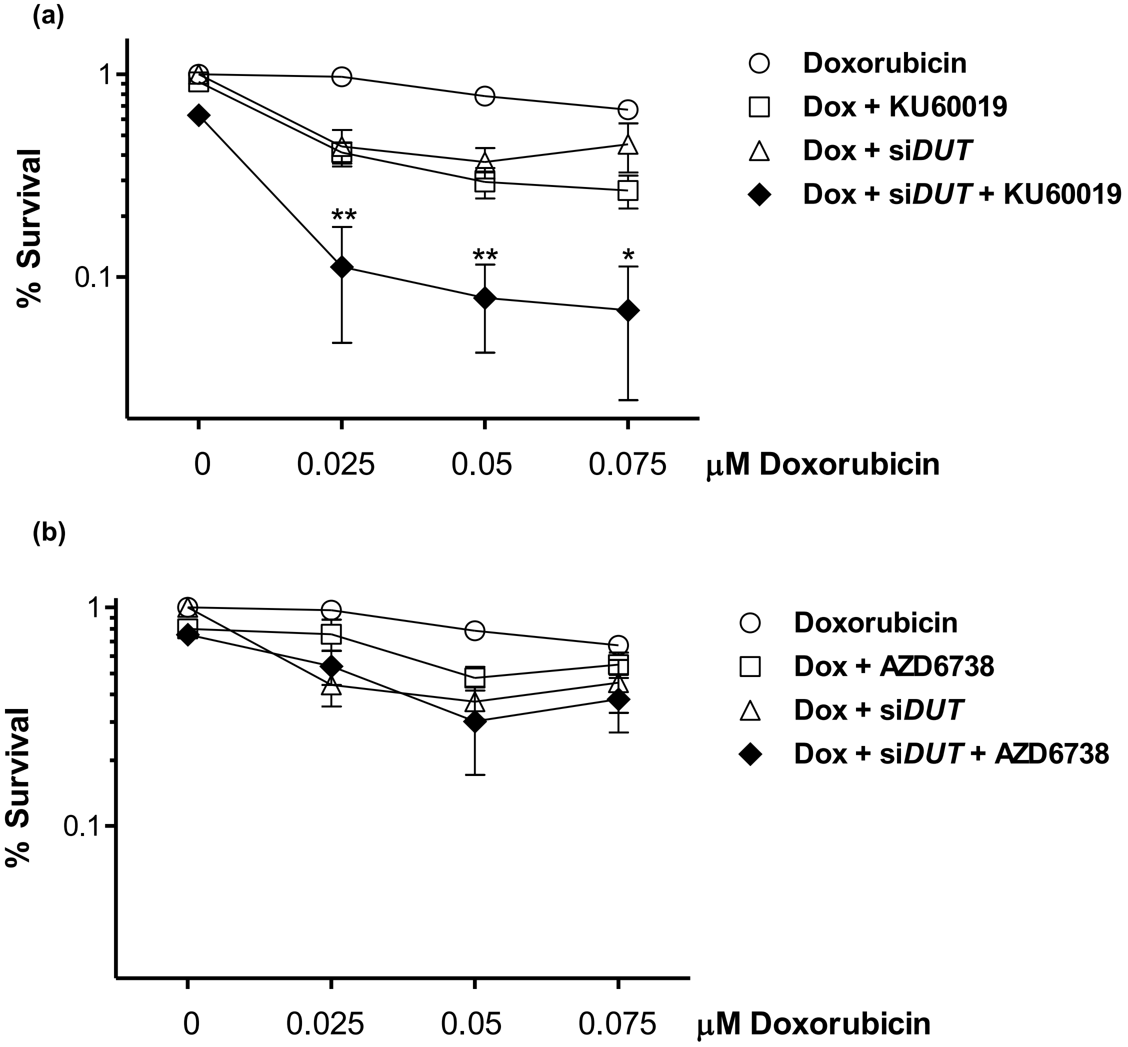

Supplementary Figure 7: dUTPase inhibition results in persistent DNA double-strand breaks following doxorubicin treatment in MDA-MB-231 cells. Cell survival was determined by colony formation assay. MDA-MB-231 cells were seeded into 24 well plates and treated with 0.025-0.075µM Doxorubicin for 4H either alone or in combination with 24H 12.5µM CV6-530 and/or (**a**) 2.5µM ATM inhibitor (KU-60019, 24H) or (**b**) 100nM ATR inhibitor (AZD6738, 24H). Drug-containing media was then replaced at the end of treatment with drug-free media. Cells were allowed to form colonies (>50 cells) for 12-15 days. All data points are expressed as mean±SEM (N=3, independent experiments). *, *P*<0.05; **, P<0.01, by an unpaired, two-tailed Student *t*-test.

SUPPLEMENTARY FIGURE 8

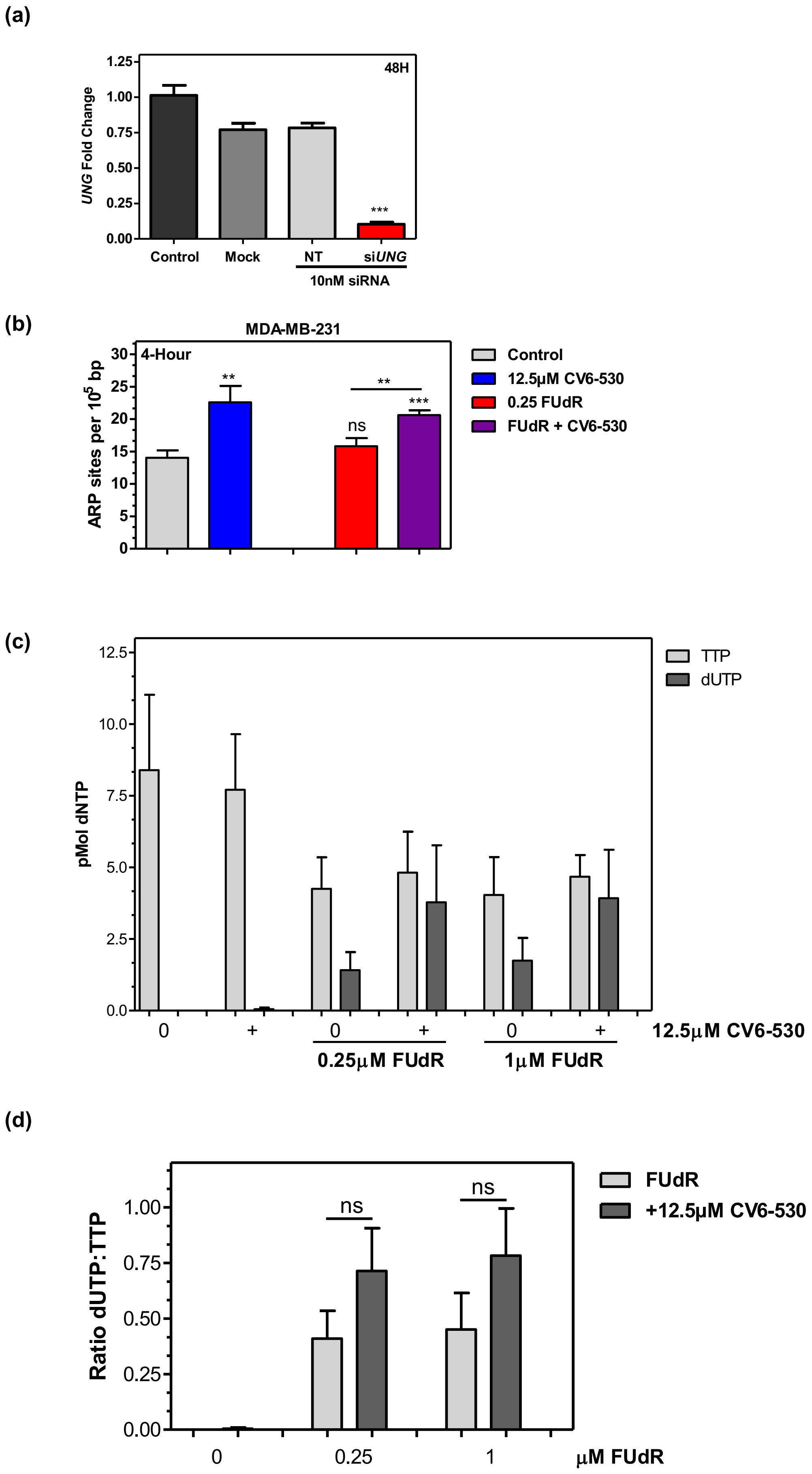

Supplementary Figure 8. Inhibition of dUTPase enhances uracil pool expansion and uracil misincorporation in MDA-MB-231 cells treated with FUdR. (a) MDA-MB-231 cells were transfected with 10nM si*UNG* and knockdown was confirmed by qRT-PCR at 48H. **(b)** MDA-MB-231 cells were transfected with 10nM si*UNG* 24H prior to treatment with 0.25μM FUdR alone or in combination with 12.5μM CV6-530. Following 4H of treatment, genomic DNA was isolated and analyzed for abasic sites using the aldehyde reactive probe (ARP) ARP assay. Data presented as the ARP sites per 10^5 bp. **(c)** MDA-MB-231 cells were treated with 0.25μM or 1μM FUdR alone or in combination with 12.5μM CV6-530 before cells were collected at 24H for quantification of intracellular TTP and dUTP levels and **(d)** the ratio of dUTP to dTTP was calculated. All data points are expressed as mean±SEM (N=3). ns, not significant; **, *P*<0.01; ***, *P*<0.001 by an unpaired, two-tailed Student *t*-test.

SUPPLEMENTARY FIGURE 9

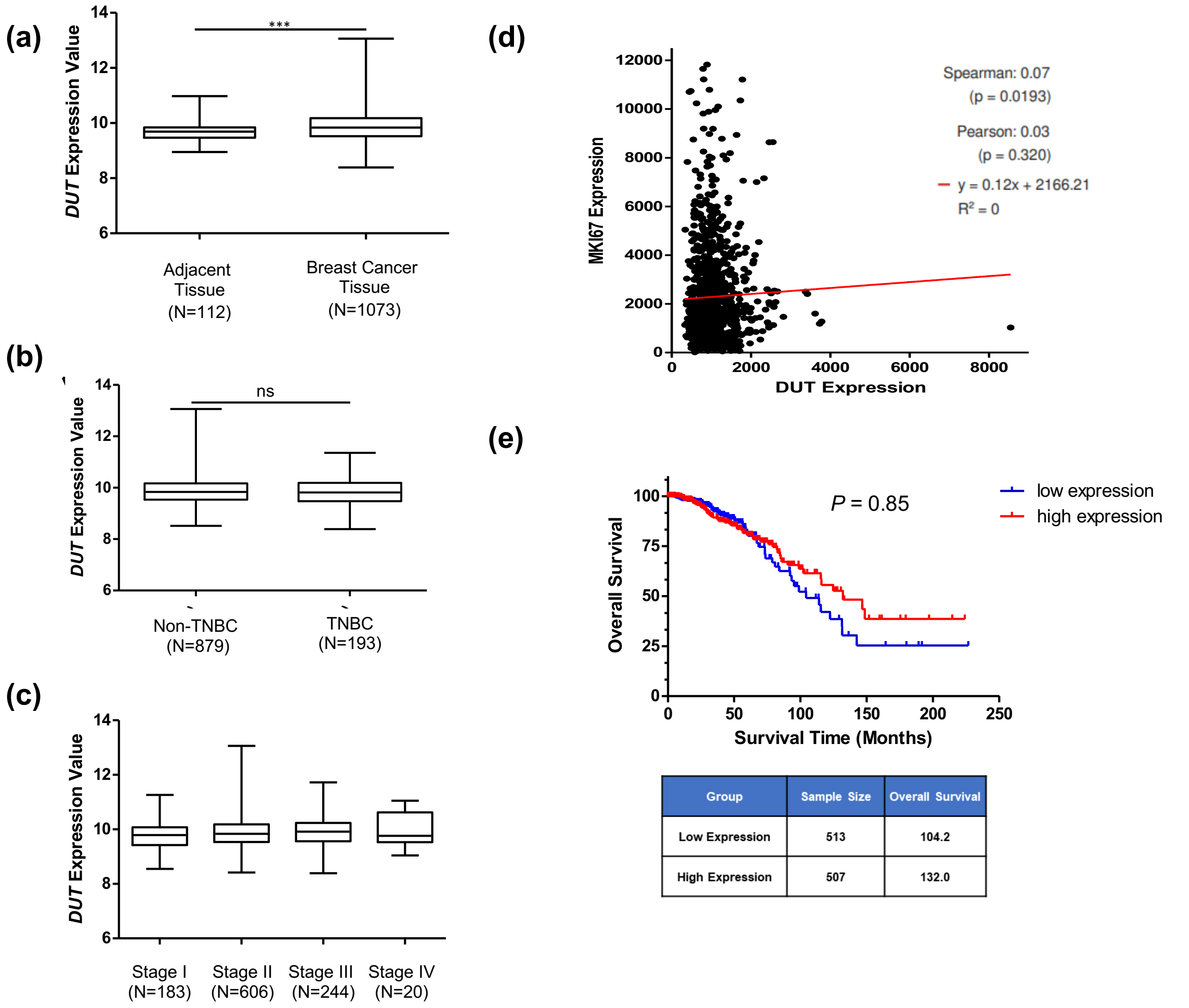

Group	Sample Size	Overall Survival
Low Expression	513	104.2
High Expression	507	132.0

Supplementary Figure 9. dUTPase expression does not correlate with overall survival for breast cancer patients from the TCGA data. Analysis was conducted on TCGA expression data from the publicly available Pan-Cancer Atlas (https://www.cell.com/pb-assets/consortium/pancanceratlas/pancani3/index.html) breast cancer data and analysed using the Breast Cancer Integrative Platform (BCIP) (http://www.omicsnet.org/bcancer/). **(a)** Box-plot comparing the mean (±SEM) expression of dUTPase (*DUT*) mRNA in breast cancer (N=1073) tissue compared to adjacent normal breast tissue (N=112) demonstrating breast cancer tissue has significantly higher *DUT* mRNA expression (two-tailed t-test, *P*=0.0005). Centre line represents the median, bounds of box represents upper and lower quartiles and whiskers represents the min and max values. **(b)** Box-plot comparing the mean (±SEM) expression of *DUT* mRNA in triple-negative breast cancer (TNBC: N=193) and non-TNBC tissues (N=879) demonstrating there was no significant difference between *DUT* expression comparing TNBC and non-TNBC breast cancer tissues (two-tailed t-test, *P*=0.91). Centre line represents the median, bounds of box represents upper and lower quartiles and whiskers represents the min and max values. **(c)** Box-plot comparing the mean (±SEM) expression of *DUT* mRNA in stage I (N=183), II (N=606), III (N=244) and IV (N=20) breast cancer tissues. There was no significant difference found between any groups, analysed by one-way analysis of variance (ANOVA). Centre line represents the median, bounds of box represents upper and lower quartiles and whiskers represents the min and max values. **(d)** Co-expression data comparing *DUT* and *MKI67* in breast cancer tissue (N=1073) demonstrated a significant positive correlation (Spearman correlation 0.07, *P*=0.0193). **(e)** Association of *DUT* mRNA expression with overall survival (OS) of all breast cancer patients with high *DUT* expression (N=507) and low *DUT* expression (N=513) being separated by median expression. No significant difference was found between OS in breast cancer patients with high or low *DUT* expression (Gehan-Breslow-Wilcoxon Test, *P*=0.85). ns, not significant; ***, *P*<0.001.

SUPPLEMENTARY FIGURE 10

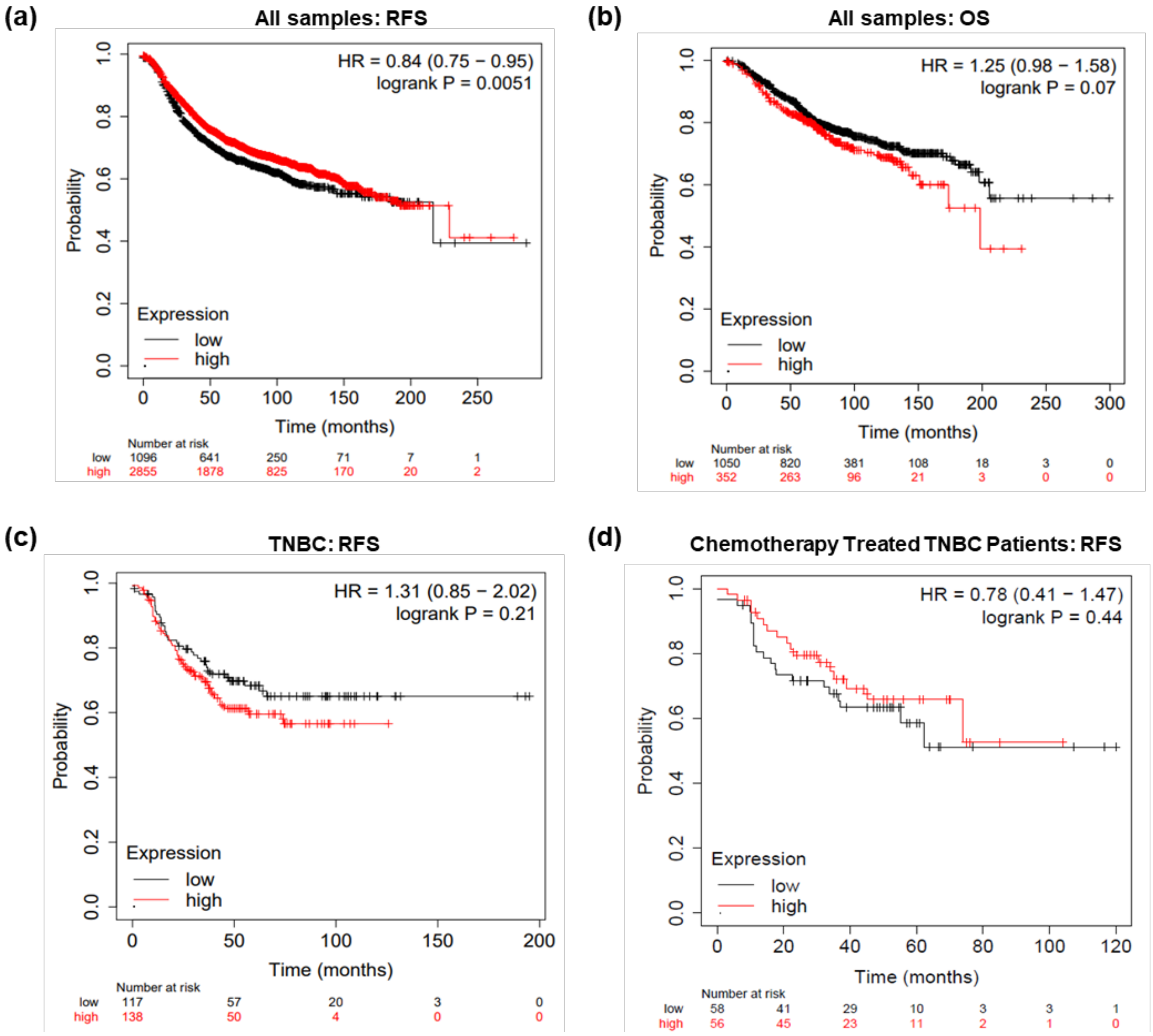

Supplementary Figure 10. Expression of dUTPase does not correlate with survival in breast cancer patients or triple negative breast cancer (TNBC) subtype. Gene expression and survival data was analysed using Kaplan-Meier Plotter (https://kmplot.com/analysis/) with data from N=3955 breast cancer patients. This software provides survival plots to assess the clinical relevance of the expression levels of various genes. Expression thresholds were set by the software selecting the cut-off with optimal performance. **(a)** Survival curve showing the association of *DUT* mRNA expression with relapse free survival (RFS) of all breast cancer patients with N=1093 patients in the low expression group and N=2855 patients in the high expression group. A significant difference (*P*=0.0051) was found between groups with patients with higher expression having better RFS resulting in a hazard ratio (HR) of 0.84. **(b)** Survival curve showing the association of *DUT* mRNA expression with overall survival (OS) of all breast cancer patients with N=1050 patients in the low expression group and N=352 patients in the high expression group. No significant difference was found between groups (*P*=0.07) . **(c)** Association of *DUT* mRNA expression with RFS of triple negative breast cancer (TNBC) patients with N=117 patients in the low expression group and N=138 patients in the high expression group. No significant difference was found between groups (*P*=0.21). **(d)** Association of *DUT* mRNA expression with RFS of TNBC patients who received chemotherapy treatment. There were N=58 patients in the low expression group and N=56 patients in the high expression group and no significant difference (*P*=0.44) was found between groups.

SUPPLEMENTARY TABLE 1

Supplementary Table 1. Experimental siRNA sequences

Gene	Company	Catalog No.	siRNA sequence
DUT	Dharmacon	M-011795-00	SMARTpool mixture of 4 siRNA sequences
UNG	Dharmacon	M-010258-01	SMARTpool mixture of 4 siRNA sequences

Supplementary Table 2. qRT-PCR primer-probe sets.

Gene	Company	Assay ID:	Lot No:
DUT	Roche RealTIme Ready	125280	0000058963
UNG	Roche RealTIme Ready	110850	110850
ACTIN	Roche RealTIme Ready	143636	0000057270
