# Supplementary material for: Targeting nucleotide metabolism enhances the efficacy of anthracyclines and anti-metabolites in triple-negative breast cancer
Source: NPJ Breast Cancer. 2021 Apr 6;7:38. doi: 10.1038/s41523-021-00245-5 (PMC8024381; doi:10.1038/s41523-021-00245-5)
Supplement: Supplementary file 2 — Reporting Summary Checklist [file 41523_2021_245_MOESM2_ESM.pdf]

Corresponding author(s): Ladner RD
LaBonte MJ

Last updated by author(s): Feb 3, 2021

Reporting Summary

Nature Research wishes to improve the reproducibility of the work that we publish. This form provides structure for consistency and transparency in reporting. For further information on Nature Research policies, see our Editorial Policies and the Editorial Policy Checklist.

Statistics

For all statistical analyses, confirm that the following items are present in the figure legend, table legend, main text, or Methods section.

n/a	Confirmed	
☐	☒	The exact sample size (n) for each experimental group/condition, given as a discrete number and unit of measurement
☐	☒	A statement on whether measurements were taken from distinct samples or whether the same sample was measured repeatedly
☐	☒	The statistical test(s) used AND whether they are one- or two-sided<br>*Only common tests should be described solely by name; describe more complex techniques in the Methods section.*
☒	☐	A description of all covariates tested
☒	☐	A description of any assumptions or corrections, such as tests of normality and adjustment for multiple comparisons
☐	☒	A full description of the statistical parameters including central tendency (e.g. means) or other basic estimates (e.g. regression coefficient) AND variation (e.g. standard deviation) or associated estimates of uncertainty (e.g. confidence intervals)
☐	☒	For null hypothesis testing, the test statistic (e.g. F, t, r) with confidence intervals, effect sizes, degrees of freedom and P value noted<br>*Give P values as exact values whenever suitable.*
☒	☐	For Bayesian analysis, information on the choice of priors and Markov chain Monte Carlo settings
☒	☐	For hierarchical and complex designs, identification of the appropriate level for tests and full reporting of outcomes
☒	☐	Estimates of effect sizes (e.g. Cohen's d, Pearson's r), indicating how they were calculated

Our web collection on statistics for biologists contains articles on many of the points above.

Software and code

Policy information about availability of computer code

Data collection	Nikon Eclipse Ti-S and G:BOX Chemi XX6 (Syngene) were used to acquire images.
Data analysis	Images were analyzed using NIS elements 4.20 and Image J software.

For manuscripts utilizing custom algorithms or software that are central to the research but not yet described in published literature, software must be made available to editors and reviewers. We strongly encourage code deposition in a community repository (e.g. GitHub). See the Nature Research guidelines for submitting code & software for further information.

Data

Policy information about availability of data

All manuscripts must include a data availability statement. This statement should provide the following information, where applicable:

- Accession codes, unique identifiers, or web links for publicly available datasets
- A list of figures that have associated raw data
- A description of any restrictions on data availability

The authors declare that the data supporting the findings of this study are available within the paper and its supplementary information file. The data generated and analysed during this study are described in the following figshare data record: https://doi.org/10.6084/m9.figshare.1404595132. All the data underlying figures 1-8 of the related article, including cell survival, growth inhibition, DNA damage analysis, apoptosis, nucleotide pool and in vivo data, along with data underlying supplementary figures 1, 2, 3, 4, 5, 7, 8 and Western Blot images for supplementary figure 1a-b, are openly available as part of the figshare data record.

Field-specific reporting

Please select the one below that is the best fit for your research. If you are not sure, read the appropriate sections before making your selection.

☒ Life sciences ☐ Behavioural & social sciences ☐ Ecological, evolutionary & environmental sciences

For a reference copy of the document with all sections, see nature.com/documents/nr-reporting-summary-flat.pdf

Life sciences study design

All studies must disclose on these points even when the disclosure is negative.

Sample size	Groups of 5-6 mice for control and experimental conditions were used. Power calculations were used to determine sample size.
Data exclusions	No data was excluded from the performed analyses.
Replication	All in vitro experiments were replicated on three independent occassions and each experiment had two or three intra-experimental replicates. All in vivo studies had a minimum of 5 animals per group.
Randomization	For in vivo analysis: when tumours reached approx 100mm^3, mice were randomized to treatment groups.
Blinding	For in vivo analysis: During treatment blinding was not performed, but during measurements the investigator was blinded to the treatment group.

Reporting for specific materials, systems and methods

We require information from authors about some types of materials, experimental systems and methods used in many studies. Here, indicate whether each material, system or method listed is relevant to your study. If you are not sure if a list item applies to your research, read the appropriate section before selecting a response.

Materials & experimental systems

n/a	Involved in the study	
☐	☒	Antibodies
☐	☒	Eukaryotic cell lines
☒	☐	Palaeontology and archaeology
☐	☒	Animals and other organisms
☒	☐	Human research participants
☒	☐	Clinical data
☒	☐	Dual use research of concern

Methods

n/a	Involved in the study	
☒	☐	ChIP-seq
☒	☐	Flow cytometry
☒	☐	MRI-based neuroimaging

Antibodies

Antibodies used	pH2A.XS139 (1:5000, JBW301; Millipore), 53BP1 (1:3000, NB100-304; Novus Biologicals), goat anti-mouse IgG-HRP (1:2000, A32723; Santa Cruz) and goat anti-rabbit IgG-AP (1:2000, A32723; Santa Cruz) were utilized. For immunoblots, dUTPase (1:500, HPA054422; Sigma-Aldrich), β-Actin (1:5000, A4700; Sigma-Aldrich), anti-mouse IgG, HRP-linked (1:2000, 7076; Cell Signaling Technology) and anti-rabbit IgG, HRP-linked (1:2000, 7074; Cell Signaling Technology)
Validation	All the used antibodies are commercially available and have been validated by the following manufactures: Millipore, Novus Biologicals, Sigma-Aldrich, Cell Signalling Technology and Santa Cruz. Validation reports can be found on their websites using the catalog number indicated above.

Eukaryotic cell lines

Policy information about cell lines

Cell line source(s)	MDA-MB-231 (human, ATCC HTB-26)<br>MDA-MB-468 (human, ATCC HTB-132)
Authentication	The cell lines were purchased from vendor listed above. No further authentication was conducted.
Mycoplasma contamination	All cell lines were routinely screened for mycoplasma using the Lonza Mycoplasma Detection Kit and all experiments carried out were preformed in mycoplasma-free cells.

Commonly misidentified lines (See ICLAC register)	No commonly misidentified cell lines were used.

Animals and other organisms

Policy information about studies involving animals; ARRIVE guidelines recommended for reporting animal research

Laboratory animals	Female Balb/c mice; 4-6 weeks old; purchased from Envigo.
Wild animals	No wild animals were used in this study.
Field-collected samples	No field-collected samples were used in this study.
Ethics oversight	The mouse work was performed under an approved UK project license and approved by the institutional Queen's University Belfast Animal Welfare Ethical Review Body (AWERB).

Note that full information on the approval of the study protocol must also be provided in the manuscript.
